# Robust method for identification of prognostic gene signatures from gene expression profiles

**DOI:** 10.1038/s41598-017-17213-4

**Published:** 2017-12-05

**Authors:** Woogwang Sim, Jungsul Lee, Chulhee Choi

**Affiliations:** 1 Department of Bio and Brain Engineering, KAIST, Daejeon, 34141 Republic of Korea; 2 Cellex Life Sciences Incorporated, Daejeon, 34051 Republic of Korea

## Abstract

In the last decade, many attempts have been made to use gene expression profiles to identify prognostic genes for various types of cancer. Previous studies evaluating the prognostic value of genes suffered by failing to solve the critical problem of classifying patients into different risk groups based on specific gene expression threshold levels. Here, we present a novel method, called iterative patient partitioning (IPP), which was inspired by the receiver operating characteristic (ROC) curve, is based on the log-rank test and overcomes the threshold decision problem. We applied IPP to analyze datasets pertaining to various subtypes of breast cancer. Using IPP, we discovered both novel and well-studied prognostic genes related to cell cycle/proliferation or the immune response. The novel genes were further analyzed using copy-number alteration and mutation data, and these results supported their relationship with prognosis.

## Introduction

Genes that are related to clinical outcomes are called prognostic genes. Prognostic genes can be used to predict patient survival^1^ and uncover the molecular mechanisms of disease^2,3^. Furthermore, novel treatments may be developed by using prognostic genes as drug targets^4,5^. Due to their potential, as well as recent advances in high-throughput technologies, prognostic genes related to cancer have been unremittingly studied during the last decade^6–11^. However, the results of only a few studies have informed clinical applications^12^, as their reliability has been questioned due to the limited robustness of independent datasets and differences among results^13^.

Previously, two main approaches have been used to search for cancer prognostic genes. One approach focused on the properties of individual genes^6–8^, and the other relied on biological networks^9–11^. For example, Van de Vijver *et al*.^6^ identified 70 breast cancer prognostic genes based on the association between gene expression profiles and prognosis. Following this pioneering work, several studies^7,8^ used similar approaches to further research in this area. However, individual gene-based studies have limitations. It is difficult to ascertain the biological significance of prognostic genes, and there is a lack of consistency among studies with respect to the prognostic genes identified. To overcome these limitations, researchers have paid special attention to biological network-based approaches. Starting from a protein-protein interaction (PPI) network^9^, other biological networks, such as signaling, metabolic, and gene regulatory networks, have been approached for identification of prognosis-related networks. These biological network-based studies also have disadvantages in terms of finding new prognostic candidates from among genes with functions that have not been studied thoroughly^14^.

The log-rank test is a statistical test commonly used to determine the prognostic significance of an attribute^15^. To evaluate this, the log-rank test can be used to compare event occurrence probabilities among groups of patients stratified by risk. The choice of risk threshold value is a significant issue in this type of analysis, as there is controversy regarding the threshold value that should be used to classify patients^16^. Measures such as the median and average, and some algorithms such as StepMiner, have been used to classify patients into distinct risk groups^17–20^. Although these measures may produce suitable thresholds, other measures have the potential to yield superior thresholds. Therefore, it is not sufficient to determine the prognostic relevance of genes by analyzing differences in prognosis among patients grouped according to specific risk thresholds, such as median or average values. However, previous researchers have been unable to consider the possible significance of various thresholds.

To resolve this issue, we propose a novel approach, called iterative patients partitioning (IPP), to calculate the degree of association between prognosis and individual gene expression by exploiting the mechanism underlying the receiver operating characteristic (ROC) curve^21^. Sensitivity and specificity, which are two important measures used to assess the performance of a classification method, are interlinked; therefore, it is difficult to determine a combination of sensitivity and specificity that is representative of classification performance. With the ROC curve, all combinations of sensitivity and specificity are used to calculate a representative classification performance value. IPP also combines all log-rank test results by varying the thresholds for classifying patients into two groups; this avoids the controversial issue of selecting a particular threshold, as occurs with the log-rank test. Compared with the log-rank test, IPP exhibited better consistency for independent datasets, as well as higher sensitivity when searching for prognostic genes. Using IPP, we also identified prognostic genes specific for subtypes of breast cancer that are differentiated by receptor type, i.e., luminal (ER+/PR+), HER2-enriched (ER-/PR-/HER2+), and triple-negative (ER-/PR-/HER2-)^22^. Some of the prognostic genes identified were well known, such as cell cycle-related genes that have been previously shown to have prognostic value. Furthermore, we identified novel prognostic genes with unknown functions, and investigated their relationships with prognosis by analyzing copy-number alteration (CNA) and mutations using independent data. These results demonstrate the utility of IPP for identifying prognostic genes based on gene expression profiles.

## Results

### Characteristics of the IPP score

**Figure 1:**
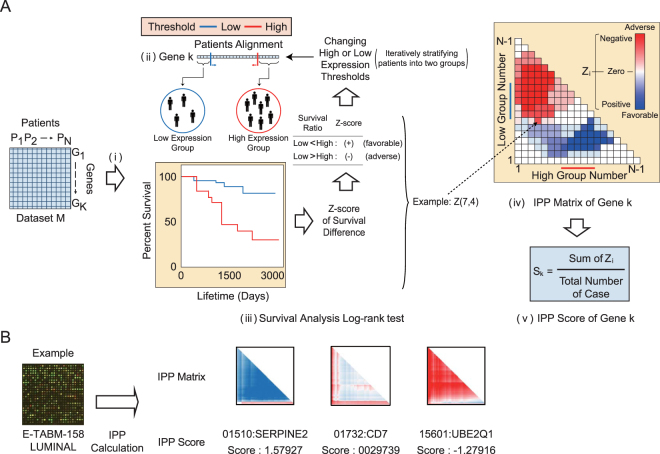
Calculation of iterative patient partitioning scores. (**A**) Schematic overview of the iterative patient partitioning (IPP) score calculation. To calculate the IPP score of a gene, patients within the individual datasets are first sorted based on gene expression level (Fig. 1A. i). Next, the patients are iteratively stratified into high and low gene expression level groups while varying the gene expression cutoff thresholds (Fig. 1A. ii), and a z-score for the survival difference between the two groups is calculated for every case, as in the log-rank test (Fig. 1A. iii). The IPP matrix is constructed using the z-scores for individual genes. In each case, the sign of the z-score is negative if patients with high gene expression levels had a higher risk of event occurrence than patients with low gene expression levels; otherwise, the z-score is positive (Fig. 1A. iv). Finally, the IPP score is the average of all z-scores (Fig. 1A. v). In Fig. 1A, the z-score calculation for seven high expression patients and four low expression patients is provided as an example. (**B**) An example IPP matrix for the luminal subtype: results from the E-TABM-158 dataset with IPP scores of 1.57927 for SERPINE2, 0.0029739 for CD7, and −1.27916 for UBE2Q1.

In a conventional log-rank test, patients are classified into two or more groups based on their gene expression levels, and the relationship between prognosis and the expression level of an individual gene is analyzed. Average and median levels of gene expression are both used as threshold values to classify patients into groups. To evaluate the prognostic significance of a gene, we designed IPP to consider all of the possible thresholds for classifying patients into two groups based on gene expression levels (Fig. 1A). The calculated z-scores of all thresholds were displayed as a matrix (IPP matrix), and the average of all z-scores was the IPP score. The sign of the IPP score represents the relationship between prognosis and gene expression level. Negative and positive IPP scores indicate adverse and favorable genes, respectively, meaning that a high level of expression is associated with a poor and good prognosis, respectively. A detailed explanation of the IPP score calculation is provided in the “Overview of the IPP calculation” section within the Materials and Methods (see Eq. (1)).

An important characteristic of the IPP score is its ability to identify the prognostic significance of genes that are missed with the conventional log-rank test, which uses average or median values as gene expression thresholds. We identified prognostic genes that were shown to be statistically insignificant by the log-rank test, using average or median threshold values, based on IPP score differences among genes (Fig. 1B). Detailed examples illustrating the differences between IPP and the conventional log-rank test are provided in Supplementary Figure 1. These examples show that IPP obtain superior results to the conventional log-rank test in terms of identifying prognostic genes. We calculated IPP scores for 21 breast cancer datasets that included a total of 2,735 patients (Table S1). To investigate IPP score characteristics, we compared the IPP score distributions among the independent breast cancer datasets (Supplementary Fig. 2 and 5A; Table S2). Despite the distributions of IPP scores being dissimilar among datasets, all datasets had a maximum frequency of over 1,500 genes, suggesting that more than half of the total genes were unrelated to prognosis.

### Robustness and consistency of IPP

**Figure 2:**
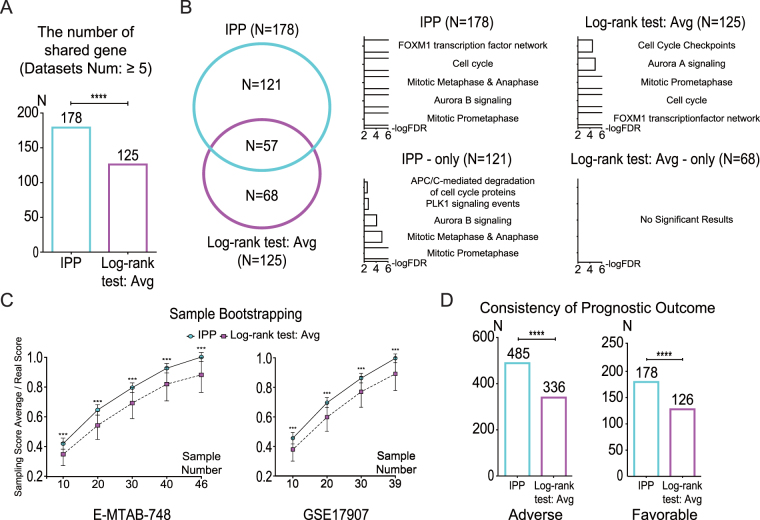
IPP identified prognostic genes more robustly than conventional log-rank test. (**A**) The number of prognostic genes shared among independent datasets, as identified by IPP and the log-rank test with the average value used as the gene expression level threshold. A gene is considered to be prognostic if its absolute score (IPP score in IPP or z-score in the log-rank test) is within the top 5% of genes, with the same number of genes used for IPP and the log-rank test to ensure a fair comparison of the genes shared among datasets. Prognostic genes shared among five or more datasets are represented by bar graphs. (**B**) Venn diagram (left) showing the number of genes detected only by IPP, only by the log-rank test, and by both methods. No functional groups were found for genes identified only by the log-rank test in Reactome pathway enrichment analysis (right). (**C**) The IPP scores represented more reliable when they were compared to the scores from subsamples versus the log-rank test. For each subsample on the x-axis, 1,000 repeated bootstrapping iterations were performed and the averaged values were plotted. In the case of the log-rank test, the z-score was used instead of a p-value. Error bars, mean ± SD. (**D**) Outcome relation (adverse or favorable) of the genes was determined more consistently by IPP than by the log-rank test. The numbers shown on the bar graphs correspond to the numbers of genes showing identical outcome relation (adverse or favorable) among 17 or more datasets. Cyan and purple colors indicate the results of IPP and the log-rank test, respectively, using the average value as the gene expression threshold. “Log-rank test: Avg” indicates the results obtained using the average value as the threshold in the log-rank test.

Robustness and consistency in prognostic gene selection refer to the degree of similarity among the prognostic genes identified from independent datasets. To verify improvement of robustness and consistency, we compared prognostic genes identified by IPP with those obtained from the conventional log-rank test, which uses the median and average values as gene expression level thresholds. First, we focused on the robustness of individual scores within each dataset by investigating the degree of dependence of each score on the number of patient samples. Bootstrapping and subsampling of two datasets, E-MTAB-748 and GSE17907, were performed and we compared the scores from all samples to those from randomly selected subsamples. IPP showed smaller differences between scores from all samples versus random subsamples than the log-rank test. In addition, variation among scores using IPP was less pronounced compared with that seen with the log-rank test (Fig. 2C and Supplementary Fig. 6C,E). We also analyzed the effect of sample size by calculating Pearson correlation coefficients (r) between the scores from all samples and those from random subsamples. IPP showed a higher correlation in all cases of subsampling than the conventional log-rank test (Supplementary Fig. 7A). These results indicate the superiority of IPP in terms of robustness compared with the conventional log-rank test.

Next, we investigated the consistency of IPP and the conventional log-rank test by comparing prognostic genes that were identified in multiple datasets. For both IPP and the log-rank test, we considered the top 5% of genes as prognostic genes, and selected the same number of genes from each dataset. The number of shared prognostic genes among several independent datasets represented the consistency. Prognostic genes shared among at least five datasets were counted (Fig. 2A and Supplementary Fig. 6A). IPP (n = 178) found significantly more shared prognostic genes than the conventional log-rank test using average (n = 125) and median (n = 135) values as gene expression level thresholds. Among the prognostic genes obtained by IPP and the conventional log-rank test, some (overlap between IPP and average: n = 57, median: n = 56) were shared between both methods, but the rest of them were not (only IPP: n = 121 vs only average: n = 68, only IPP: n = 122 vs only median: n = 79) (Fig. 2B and Supplementary Fig. 6B). We suspected that the 68 and 79 genes identified using the average and median value thresholds, respectively were likely to be false-positives and, therefore, unlikely to represent functional groups. We then performed pathway enrichment analysis using the Reactome Pathway Database^23,24^ to analyze the functional characteristics of prognostic genes found using IPP and the conventional log-rank test. As expected, no significant functional group was found for prognostic genes identified only by the conventional log-rank test (Fig. 2B and Supplementary Fig. 6B). On the other hand, cell-cycle related functional groups, such as “mitotic prometaphase” and “cell cycle”, were major features of prognostic genes identified only by IPP (Fig. 2B). These results suggest that IPP identifies prognostic genes more reliably than the conventional log-rank test.

Lastly, we examined the consistency of the relationship between the expression levels of the identified genes and prognosis. A gene is considered adverse if high-level expression is associated with a poor prognosis, such as a short survival or relapse time. When high expression of a gene is associated with a good prognosis, then the gene is considered favorable. We refer to these relationships as outcome relation. Since the outcome relation is a significant feature of prognostic genes, we analyzed the consistency of outcome relation among datasets (Fig. 2D, Supplementary Fig. 6D and Supplementary Fig. 7B). Each gene is assigned two numbers, indicating the number of datasets in which the gene is adverse and favorable. To compare the consistency of outcome relation between IPP and the conventional log-rank test, the numbers of datasets in which the gene is adverse and favorable were counted for each gene (Supplementary Fig. 7B). We summed the numbers of genes that were adverse or favorable in at least 17 datasets. IPP had a greater number of genes in the adverse (n = 485) and favorable (n = 178) classes than the log-rank tests using the average (adverse, n = 336; favorable, n = 126) and median (adverse, n = 337; favorable, n = 139) values as expression level thresholds (Fig. 2D and Supplementary Fig. 6D). These results show the higher robustness and consistency of IPP than compared with the conventional log-rank test.

### Molecular subtype-specific breast cancer prognostic genes

It is well known that different subtypes of breast cancer have distinct characteristics. To investigate subtype-specific prognostic genes, we calculated IPP scores for a total of 16 breast cancer datasets by reference to three distinct molecular subtypes: luminal (ER-positive/PR-positive; Supplementary Fig. 9A), HER2-enriched (ER-negative/PR-negative/HER2-positive; Supplementary Fig. 9C), and triple-negative (ER-negative/PR-negative/HER2-negative; Supplementary Fig. 9B) based on ER, PR, and HER2/ERBB2 immunohistochemistry information. Datasets with fewer than 20 patient samples for three molecular subtypes were excluded from the analysis. To ensure that the numbers of patients for all datasets were considered, we calculated representative IPP scores for gene using Liptak’s weighted method^25^ (Supplementary Fig. 9D). Subtype-specific prognostic genes represented the top 5% of all genes (n = 557 genes among a total of 11,123 genes) based on the absolute values of the representative, integrated IPP scores. Next, we performed Reactome pathway enrichment to investigate the characteristics of subtype-specific prognostic genes.

**Figure 3:**
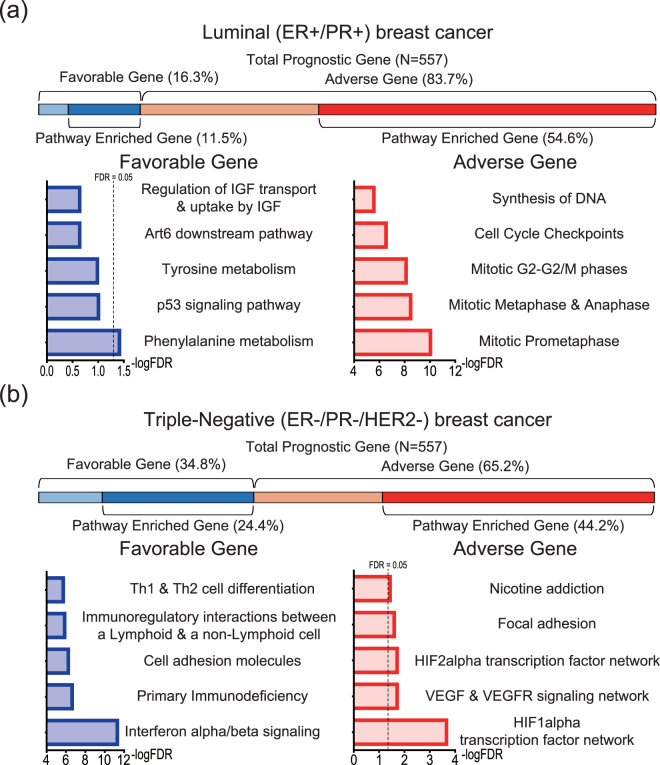
Subtype-specific functional groups of prognostic genes. (**a**) Luminal (ER-positive/PR-positive) specific prognostic genes and functional groups. In total, 83.7% and 16.3% of prognostic genes (n = 557) were adverse and favorable, respectively; 54.6% and 11.5% of the adverse and favorable prognostic genes, respectively, were assigned to functional groups through Reactome pathway enrichment analysis (upper panel). (**b**) Triple-negative (ER-negative/PR-negative/HER2-negative)-specific prognostic genes and functional groups: 65.2% and 34.8% of the prognostic genes (n = 557) were adverse and favorable, respectively. In total, 44.2% and 24.4% of the adverse and favorable prognostic genes, respectively, were assigned to Reactome functional groups (upper panel). Representative functional groups of adverse (red) and favorable (blue) prognostic genes (lower panel). The colors of the bars in the figure indicate the outcome relation (adverse or favorable), and the enriched and non-enriched portions of the prognostic genes. X-axis: common logarithm of the false discovery rate (FDR).

In luminal (ER-positive/PR-positive) breast cancer, the proportion of adverse and favorable genes was 83.7% (n = 466) and 16.3% (n = 91), respectively, out of a total of 557 prognostic genes (Fig. 3a, upper). Based on enrichment analysis, only 54.6% (n = 304) of adverse genes and 11.5% (n = 64) of favorable genes were included within the functional groups. Enrichment analysis showed that adverse genes were composed of cell cycle-related genes, such as those involved in “mitotic prometaphase”, “mitotic metaphase and anaphase” and “cell cycle checkpoints” (Fig. 3a, lower right). In addition, 50.2% of the adverse prognostic genes (n = 234) had at least one PPI with each other, and thus serve to organize PPI networks (Supplementary Fig. 11, Middle). Among the favorable genes, we observed several functional groups, such as “phenylalanine metabolism” and “p53 signaling pathway” (Fig. 3a, lower left panel).

The results for HER2-enriched (ER-negative/PR-negative/HER2-positive) and triple-negative (ER-negative/PR-negative/HER2-negative) breast cancers differed from those for luminal (ER-positive/PR-positive) breast cancer. In HER2-enriched breast cancer, 37.7% (n = 210) of the 62.7% (n = 349) of prognostic genes that were adverse were related to extracellular matrix functions, such as “Extracellular matrix organization” and “Integrin signaling pathway” (Supplementary Fig. 10A, lower right). Fewer adverse prognostic genes (30.1%, n = 105) organized PPI networks in HER2-enriched cancer than in luminal breast cancer. In contrast, a great number of favorable prognostic genes (21.2%, n = 44) organized PPI networks in HER2-enriched cancer than luminal in breast cancer (Supplementary Fig. 12). Despite lower significance, the 25.6% (n = 143) of prognostic genes that were favorable for HER2-enriched breast cancers were mainly involved in “Keratinization”, “Wnt signaling pathway” and “Vitamin D metabolism” (Supplementary Fig. 10A, lower left panel).

In triple-negative breast cancer, 65.2% (n = 363) and 34.8% (n = 194) of prognostic genes were adverse and favorable, respectively (Fig. 3b and Supplementary Fig. 13). Among these, 44.2% (n = 246) and 24.4% (n = 136) were included in the Reactome pathway enrichment functional groups (Fig. 3b, upper panel). Unlike luminal and HER2-enriched breast cancers, pathways related to the hypoxia inducible factor, (HIF), such as “HIF-1 alpha transcription factor network” and “HIF-2 alpha transcription factor network”, were major functional features of adverse genes in triple-negative breast cancer. “VEGF & VEGFR signaling network”, “Focal adhesion”, and “Nicotine addiction” were also important functional features of adverse prognostic genes for triple-negative breast cancer (Fig. 3b, lower right panel). On the other hand, favorable prognostic genes for triple-negative breast cancer had immune-related functional features, such as “Interferon alpha/beta signaling”, “Immunoregulatory interactions between a lymphoid & a non-lymphoid cell”, and “Th1 & Th2 cell differentiation” (Fig. 3b, lower left panel). Less than 20 and 10 genes were shared in the adverse and favorable prognostic gene classes, respectively, among luminal, HER2-enriched, and triple-negative breast cancer (data not shown), suggesting remarkably distinct functional characteristics of adverse and favorable prognostic genes among the three molecular subtypes of breast cancer.

### Novel prognostic genes identified by IPP

The IPP method led to the identification of prognostic genes that had unknown functions and were therefore not assigned during enrichment analysis. We hypothesized that these genes were novel prognostic genes; therefore, we performed detailed analyses of copy number alteration (CNA) and mutations of these genes using The Cancer Genome Atlas (TCGA) data provided by cBioportal^26,27^ and the International Cancer Genome Consortium (ICGC)^28^. We analyzed CNA of 1,506 patients from METABRIC database of the TCGA^29^. A total of 906 patients with simple somatic mutations were selected from among the BRCA-US database of the ICGC. Detailed information regarding the process by which patients were grouped by CNA or mutation status is provided in the Materials and Methods. We then performed the log-rank test based on disease-free survival (DFS) and overall survival (OS).

**Figure 4:**
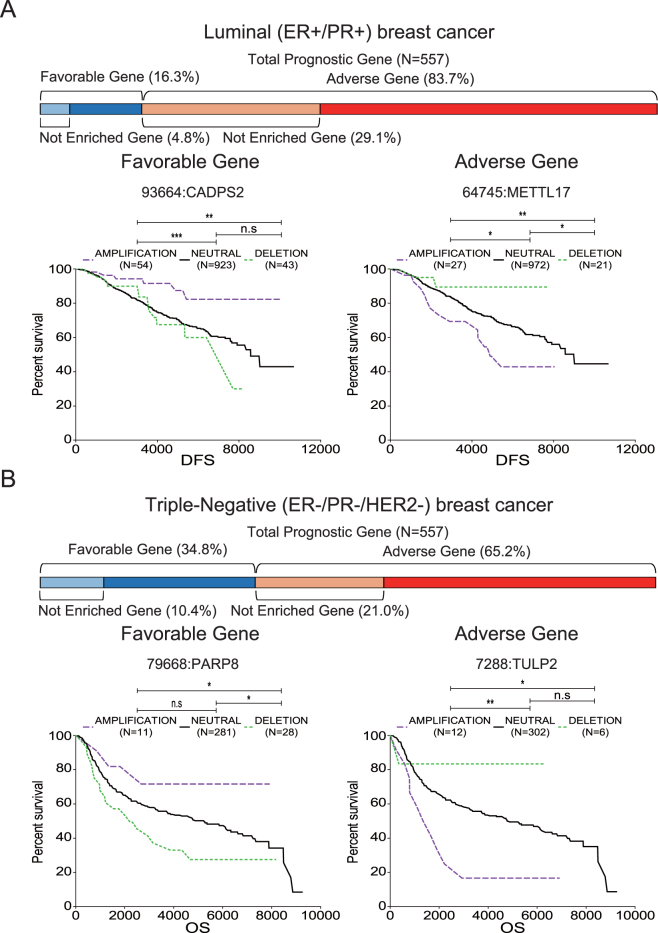
Prognostic significance of copy number alteration (CNA) in functionally unknown prognostic genes identified by IPP. (**A**) Among all prognostic genes (n = 557) for luminal breast cancer, 29.1% and 4.8% of the adverse and favorable prognostic genes, respectively, were not assigned to any functional group by Reactome pathway enrichment analysis (upper panel). Kaplan-Meier curves show the difference in disease-free survival (DFS; days) according to the CNA states (amplification, neutral, or deletion) of CADPS2 and METTL17, favorable and adverse prognostic genes, respectively, which were not assigned to any functional group by Reactome pathway enrichment analysis (lower panel). (**B**) Among all prognostic genes (n = 557) for triple-negative breast cancer, 21.0% and 10.4% of adverse and favorable prognostic genes, respectively, were not assigned to any functional group by Reactome pathway enrichment analysis (upper panel). Kaplan-Meier curves show the differences in overall survival (OS; days) according to the CNA state (amplification, neutral, or deletion) of PARP8 and TULP2, favorable and adverse prognostic genes, respectively, which were not assigned to any functional group by Reactome pathway enrichment analysis (lower panel). The colors of the Kaplan-Meier curves indicate the CNA states (purple: amplification, black: neutral, green: deletion). Each n on the Kaplan-Meier curves indicates the number of patients included in that CNA state.

Among all prognostic genes (n = 557), about 30% were not assigned to any functional group by Reactome pathway enrichment analysis of luminal (33.9%: adverse, 29.1%; favorable, 4.8%, Fig. 4A, upper panel), HER2-enriched (36.7%: adverse, 25.0%; favorable, 11.7%, Supplementary Fig. 10B, upper panel), and triple-negative (31.4%: adverse, 21.0%; favorable, 10.4%, Fig. 4B, upper panel) breast cancers. Among the unassigned genes, the CNA states of METTL17 showed a marked association with prognosis (Fig. 4A, lower right panel). Because METTL17 was an adverse prognostic gene on IPP, higher gene expression of METTL17 indicates a poorer prognosis. Therefore, it is possible that patient groups with amplification and deletion of this gene have lower and higher survival probability than the neutral group, respectively. The other three representative genes, CADPS2 (Fig. 4A, lower left panel), PARP8, and TULP2 (Fig. 4B, lower panel) showed results consistent with those obtained by IPP. Despite few patient samples showing mutations, and a lack of information on breast cancer subtypes, we found that prognostic genes identified by IPP, such as DONSON, MKI67, FAM171A1, TENM4, C16orf45, and RABEP2, showed statistically differences in survival probability between mutation and non-mutation groups (Supplementary Fig. 14). These data demonstrate that IPP has the power to identify novel prognostic genes.

## Discussion

Here, we propose a novel method for evaluating the prognostic performance of individual genes. Survival analysis compares the probabilities of events between different groups of patients divided by a value of interest. For example, patients may be divided into groups based on their levels of gene expression. The log-rank test is a method used to determine whether the difference in probability of an event’s occurrence between different groups of patients is statistically significant. However, there is no general rule on how to divide patients into groups based on gene expression levels. The average or median is typically used as a threshold value for dividing patients into two groups. Commonly used thresholds, such as median and average values^17–20^, risk misevaluation of the prognostic significance of a gene, where using a single value as the threshold may cause the significance of other values to be overlooked in survival analysis. For example, when identifying a statistically significant difference in prognosis between groups, it is possible that patients will be divided into two asymmetric groups; however, when the median value is used as the threshold, patients are divided into two symmetrical (equal) groups. In addition, use of the average value as a threshold is insufficient for determining whether a difference in prognosis exists between two groups of patients, as the average is particularly susceptible to the influence of outliers. Thus, we can only consider a limited number of possible results of survival analysis when we use single-value thresholds, such as the median or average. Results obtained by other threshold measures are ignored in survival analysis when a single-value threshold is used. To overcome these limitations and account for as many situations as possible, we developed IPP, which utilizes all gene expression thresholds to group patients.

Genes useful for disease prognosis can theoretically be recognized in any independent dataset including samples from the disease population. Inconsistency has been a major hindrance in the search for prognostic genes^30^, especially when gene expression profiles are used. In this study, we showed that prognostic genes identified by IPP were more consistently present among independent datasets than those identified by the conventional log-rank test (Fig. 2 and Supplementary Figs 6 and 7). In addition, functional enrichment analysis showed that the prognostic genes consistently identified by IPP play roles in cancer, as evidenced by the enrichment of genes involved in the cell cycle (Fig. 2B and Supplementary Fig. 6B)^31,32^.

Representative characteristics of cancer include cell cycle modification and uncontrolled proliferation. Molecular markers related to the cell cycle are often examined in breast cancer^33^. Likewise, we observed that the cell cycle functional group was most strongly associated with prognosis in luminal (ER-positive/PR-positive) breast cancer (Fig. 3a). Despite lower significance in terms of the false discovery rate (FDR) of enriched functional groups, phenylalanine and tyrosine metabolism may be related to prognosis in patients with luminal breast cancer^34^. One of our identified functional groups, the p53 signaling pathway, is a well-studied tumor suppressing protein signaling pathway. In addition, the Atf6 downstream pathway, which plays a role in mediating apoptosis^35^ but has rarely been studied, is a reasonable target for luminal breast cancer. Even though these findings are based on an analysis of only two datasets (GSE17907 and GSE45255), some of the results, such as “Extracellular matrix organization^36^”, “Integrin signaling pathway^37^”, “PDGF receptor signaling network^38^” and “Vitamin D metabolism^39^”, seem to be significant elements in HER2-enriched breast cancer. In particular, our results support the utility of therapy targeting extracellular matrix components for HER2-enriched breast cancer, in accordance with previous research reporting that dense extracellular matrix components around the tumor could block trastuzumab (Herceptin) targeting of HER2^36^. Interestingly, in triple-negative breast cancer, HIF-related genes (HIF1alpha and HIF2alpha transcription factor network) were identified primarily as adverse prognostic genes (Fig. 3b, lower right). As in previous studies of triple-negative breast cancer^40^, this suggests that therapies targeting HIF signals may improve the prognosis of triple-negative breast cancer patients by inhibiting breast cancer progression. The “Nicotine addiction” functional group found for the adverse genes for triple-negative breast cancer may be related to breast-to-brain metastasis. All six genes are GABA receptor-related, and some previous studies have reported that an increase in neurotransmission in association with activation of GABA receptors influences breast cancer metastasis to the brain^41,42^.

We corroborated the prognostic significance of the novel genes identified by IPP to which no function was assigned by cross-checking various independent datasets. We found that differences in prognosis were statistically significant on analysis of the presence of CNA and simple somatic mutations in some genes. These results show that IPP is a robust method for assessing the prognostic relevance of genes, even though these analyses are limited by unbalanced patient numbers among groups. Regarding CNA, fewer samples had deletions compared with those that did not. Samples with mutations were also less common than those without mutations. This imbalance could weaken the statistical significance of our results; however, the prognostic relevance of functionally unknown genes identified by IPP, for example METTL17 which has been previously reported as a possible prognostic gene^43^, was cross-validated using independent datasets. Together, the results demonstrated the reliability of IPP and showed that it is a promising method for identifying prognostic factors. According to our results, patients with simple somatic mutations on both adverse and favorable prognostic genes have an overall poor prognosis. If we conceive of mutations in terms of gain- or loss-of-function, we suggest that adverse gene mutations are gain-of-function mutations, also referred to as oncogenes, the expression levels of which have a negative relationship with prognosis. For example, a simple somatic mutation of MKI67 can be classified as a gain-of-function mutation based on our results, and the findings of a previous study showing that MKI67 is a proliferation marker^44^. Mutations in favorable genes may cause loss-of-function, and are thus considered tumor suppressor genes.

In conclusion, we developed IPP, a powerful method to search not only for well-known prognostic genes, but also for promising novel prognostic genes. We used IPP to analyze the relationship between prognosis and gene expression. Furthermore, IPP can be widely applied to analyze the relationships of other data types with prognosis, as long as we can classify patients according to those data. Therefore, we believe that IPP will lead to the identification of factors that can be used to predict disease prognosis, develop novel drugs, and study disease mechanisms.

## Materials and Methods

### Data sources

Nineteen breast cancer microarray datasets of breast cancer containing clinical survival information were used in this study. Sixteen of the datasets were downloaded from the Gene Expression Omnibus (GEO) database of the National Center for Biotechnology Information (NCBI), operated by the US National Library of Medicine (http://www.ncbi.nlm.nih.gov/geo/). The other three datasets were downloaded from ArrayExpress of the European Bioinformatics Institute, run by the European Molecular Biology Laboratory (https://www.ebi.ac.uk/arrayexpress/). Because the GSE6532 dataset includes data from multiple hospitals, we divided it into three subsets according to the hospital and microarray platform. In total, 21 independent breast cancer datasets were used in this study, normalized according to the default options of the SCAN.UPC package of BioConductor/R (Table S1). We used Entrez Gene ID (ver. 17.0; BrainArray) to summarize the probes and then converted the data to HPRD protein ID (ver 9.0) data. The normalized data were reported previously (https://www.oncopression.com)^45^. Data from TCGA (METABRIC)^29^, which contain information on individual patients, were downloaded from the cBioportal for Cancer Genomics operated by Memorial Sloan-Kettering Cancer Center (http://www.cbioportal.org/)^26,27^. We also collected BRCA-US data (release 22) from the ICGC, which originates from the United States (https://dcc.icgc.org)^28^.

### Study subject selection

Based on survival information, 2,735 breast cancer patients were selected among the 21 microarray datasets as the study subjects. The patients in each dataset were excluded from the analysis if they were not cancer patients. Subjects without distant metastasis-free survival (DMFS) information were also excluded. To avoid errors, we only used data from subjects with event and time DMFS data in our calculations (Table S1). In total, data from 1,020 luminal breast cancer subjects, 134 HER2-enriched breast cancer subjects, and 320 triple-negative breast cancer subjects with OS and DFS information were selected from METABRIC dataset in cBioportal database. We also included the data of 906 breast cancer subjects within the BRCA-US data of the ICGC with “Primary tumour - solid tissue” as the specimen type and a “donor interval of last follow up” time greater than zero.

### Overview of the IPP calculation

An IPP matrix corresponds to the prognostic signature of a single gene within a microarray dataset. Each element of the matrix represents an independent case, which partitions patient samples according to gene expression level and survival information. Given a microarray dataset M, we can assume that K number of genes and N number of patient samples are included. In the case of the k^th^ gene, patient samples were aligned based on the corresponding gene expression level. After patient sample alignment, the samples were partitioned into high and low gene expression level groups. Before partitioning the patient samples, we filtered out the genes with nearly identical expression levels among the majority of patient samples. Patient samples with gene expression levels that were lower than the low group threshold were classified into the low group. Similarly, patient samples with gene expression levels higher than the high group threshold were classified into the high group. Each threshold was changed iteratively, with no overlap of samples between groups. Thus, the number of patient samples in each group could not exceed n-1. The samples were partitioned iteratively by changing the low and high group thresholds for each element of the matrix. We applied log-rank statistics to determine if survival probability was statistically different between the high and low groups. The z-scores from the log-rank test were used to represent each element in the matrix. A negative z-score indicated that patients in the high group had a higher risk of cancer versus those in the low group. Finally, the IPP score was calculated as the average of all z-scores in the IPP matrix. For the k^th^ gene, the gene score S_k_ was defined as:

1 $${{\rm{S}}}_{{\rm{k}}}=\frac{1}{\frac{{\rm{N}}({\rm{N}}-1)}{2}}\times \sum _{{\rm{i}}={\rm{1}}}^{{\rm{N}}-{\rm{1}}}\sum _{{\rm{j}}={\rm{1}}}^{{\rm{N}}-{\rm{i}}}{Z}_{{\rm{ij}}},$$

where *i* and *j*, are indexes of the high and low group thresholds, respectively, and Z_ij_ is the score of the (i, j) matrix element. The negative and positive IPP scores reflect adverse and favorable outcomes, respectively (Fig. 1A). The distributions of all calculated IPP scores from among the 21 breast cancer datasets are presented as supplementary data (Supplementary Figs 2, 5 and 9). Genes that present in all datasets (n = 11, 123) were analyzed in this study.

### Calculation of the random IPP score distribution: arbitrary patient sampling

To determine the distribution of the random IPP score, we calculated the IPP scores 10,000 times for each number of random patient samples. First, we created patient samples virtually with randomly selected survival events and survival times. We performed 10,000 times calculation of IPP scores for 10 different number of random patient samples, involving 20 to 250 random patient samples. The IPP score distributions generated by the arbitrary patient sampling were organized based on the results of 10,000 calculations (Supplementary Fig. 8).

### Comparison between IPP and average/median thresholds

To compare the robustness of the IPP results with those of the conventional log-rank test, we computed z-scores using the log-rank test according to average (Supplementary Fig. 3) and median (Supplementary Fig. 4) gene expression level threshold values. After filtering out genes with nearly identical expression levels among all patient samples, we compared the IPP and conventional log-rank test results for 11,123 genes (Fig. 2 and Supplementary Figs 6 and 7).

For the 11,123 total genes, we compared the number of shared genes among the datasets obtained by IPP and the log-rank test according to average (Fig. 2A,B) and median (Supplementary Fig. A,B) value expression level thresholds. To determine the number of genes shared among datasets using the IPP and log-rank test according to the average and median thresholds, the top 5% of genes, based on absolute IPP score and z-score, were first filtered from the datasets. Shared genes were those that were filtered from at least 5 datasets.

We assessed the consistency of outcome relation (adverse or favorable) among the different datasets (Fig. 2D, Supplementary Fig. 6D and Supplementary Fig. 7B). Among the 21 breast cancer datasets, each gene could be categorized as adverse or favorable based on its IPP score or z-score. Because the numbers of adverse and favorable cases were dependent on each other, and their sum must be 21, the total number of adverse-favorable pairs was 21. We counted the number of genes included in each pair (adverse-favorable).

In addition, bootstrapping (Fig. 2C, Supplementary Fig. 6C and Supplementary Fig. 7A) and subsampling (Supplementary Fig. 6E and Supplementary Fig. 7A) were performed. All sampling was repeated 1,000 times for each number of samples. We used only two datasets, E-MTAB-748 and GSE17907, which have smaller numbers of patient samples than other datasets. Through bootstrapping and subsampling, two different values were compared with the IPP score or the z-score produced by the log-rank test: average sampling scores and Pearson correlation coefficient, r. To avoid the issue of scaling, we compared average sampling scores that were normalized by the IPP score or the z-score produced by the log-rank test.

### PPI network and Reactome pathway enrichment

HUGO Gene Nomenclature Committee (HGNC) symbols for prognostic genes were transformed into the corresponding Entrez Gene symbols for Reactome pathway analysis. We used the Reactome Functional Interaction (FI) plugin and the Cytoscape program^46^ to depict PPI networks and perform pathway enrichment. The “Gene Set/Mutation Analysis” option in the Reactome FI plug-in was used to analyze prognostic genes based on “Gene set format,” with “Fetch FI annotations” and “Show genes not linked to others” conditions set within the “FI Network Construction Parameters”. Single nodes with no PPI connections were not represented within the Reactome pathway (Supplementary Figs 11–13) but were included in pathway enrichment results.

### Log-rank test of patients with CNA data from the TCGA database and mutation data from the ICGC database

We utilized patient data that included information on CNA, OS, and DFS. Patients were classified into five CNA groups using TCGA data obtained from cBioportal: 1) homozygous deletion, 2) hemizygous deletion, 3) neutral/no change, 4) gain, and 5) high-level amplification. To ensure a sufficient number of patient samples in each CNA group, we divided patients according to three categories: 1) homo/hemizygous deletion to deletion, 2) neutral/no change to normal and 3) gain/high-level amplification to amplification. A one-sided log-rank test was performed.

The data of 906 breast cancer patients, extracted from the BRCA-US dataset of the ICGC, were used to determine the significance of simple somatic mutations in terms of prognosis. The survival data of each patient subsumed four different attributes: donor vital status, disease status last follow-up, donor survival time, and donor interval of last follow up. The “donor vital status” of “deceased” and “alive” were classified as “event occurred” and “event did not occur” in event OS, respectively. The values for “donor survival time” and “donor interval of last follow-up” corresponded to the time OS for “deceased” and “alive” patients, respectively. To assess the influence of simple somatic mutations on the prognosis of breast cancer patients, we divided patients into mutation and non-mutation groups based on “consequence type” and “amino acid mutation” criteria. Patients with any “consequence type” mutations of a gene were included in the “mutation” group; and all other patients were included in the “none” group. A one-sided log-rank test was performed.

### Statistical analysis

A one-tailed *z*-test, based on a binomial distribution with the ratio of shared genes to total genes, was performed to analyze differences of number of shared genes among datasets between IPP and the log-rank test. A two-tailed unpaired Student’s *t*-test was used to analyze the bootstrapping and subsampling results. A two-tailed paired Student’s *t*-test was performed to determine the consistency of outcome relation among datasets. A one-sided log-rank test using CNA and mutation data was performed. FDR values from Reactome Functional Enrichment were calculated using a Reactome plugin and Cytoscape software.

### Data availability

The data from this study are available from the authors upon request.

## Electronic supplementary material

Supplementary Information
