# Supplementary material for: Robust method for identification of prognostic gene signatures from gene expression profiles
Source: Sci Rep. 2017 Dec 5;7:16926. doi: 10.1038/s41598-017-17213-4 (PMC5717170; doi:10.1038/s41598-017-17213-4)
Supplement: Supplementary file 1 — Supplementary Information [file 41598_2017_17213_MOESM1_ESM.pdf]

Robust method for identification of prognostic gene signatures from gene expression profiles

Woogwang Sim[1], Jungsul Lee[2, *] and Chulhee Choi[1, 2, *]

[1]Department of Bio and Brain Engineering, KAIST, Daejeon 34141, Republic of Korea

[2]Cellex Life Sciences Incorporated, Daejeon 34051, Republic of Korea

*Email: adnoctum@kaist.ac.kr or cchoi@kaist.ac.kr

Supplementary Information

SI Guide

Supplementary Figure 1.	IPP matrix examples.
Supplementary Figure 2.	Histogram showing IPP score distributions from 21 breast cancer datasets.
Supplementary Figure 3.	Histograms of z-score distributions from log-rank test with average threshold of gene expression level.
Supplementary Figure 4.	Histograms of z-score distributions from log-rank test with median threshold of gene expression level.
Supplementary Figure 5.	Box and whisker plots of the distributions from IPP scores and z-scores of log-rank test with average/median gene expression level threshold.
Supplementary Figure 6.	IPP identified prognostic genes more robustly than conventional log-rank test with median threshold.
Supplementary Figure 7.	Robustness comparison by sampling of Pearson correlation and outcome relation consistency between IPP and conventional log-rank test with average/median gene expression level threshold.
Supplementary Figure 8.	IPP score distributions from arbitrary, random-made patient samples.
Supplementary Figure 9.	IPP score distributions of breast cancer subtypes: Luminal (ER-positive/PR-positive), HER2-enriched (ER-negative/PR-negative/HER2-positive), and Triple-

negative (ER-negative/PR-negative/HER2-negative).

Supplementary Figure 10. Functional groups of prognostic genes from HER2-enriched (ER-negative/PR-negative/HER2-positive) breast cancer.

Supplementary Figure 11. Protein-Protein Interaction network pathway of Luminal (ER-positive/PR-positive) breast cancer.

Supplementary Figure 12. Protein-Protein Interaction network pathway of HER2-enriched (ER-negative/PR-negative/HER2-positive) breast cancer.

Supplementary Figure 13. Protein-Protein Interaction network pathway of triple-negative (ER-negative/PR-negative/HER2-negative) breast cancer.

Supplementary Figure 14. Prognostic association of mutation of functionally unknown prognostic genes identified by IPP.

Supplementary Table 1. Breast Cancer Microarray Datasets

Supplementary Table 2. Breast Cancer Microarray Datasets - IPP Score Statistics

Supplementary Table 3. Breast Cancer Microarray Datasets - Z-Score Statistics from log-rank test with average threshold

Supplementary Table 4. Breast Cancer Microarray Datasets - Z-Score Statistics from log-rank test with median threshold

Supplementary Table 5. Arbitrary, random-made Patients Sampling Score Distributions - Statistics

Supplementary Table 6. Luminal (ER-positive/PR-positive) Breast Cancer - IPP Score Statistics

Supplementary Table 7. HER2-enriched (ER-negative/PR-negative/HER2-positive) Breast Cancer - IPP Score Statistics

Supplementary Table 8. Triple-negative (ER-negative/PR-negative/HER2-negative) Breast Cancer - IPP Score Statistics

References

Supplementary Figure 1. Example IPP matrices.

The z-score distributions of IPP matrices from four different genes within the GSE2034 dataset are indicated by A, E, I, and M. The x- and y-axes of the matrix represent the number of subjects in the high and low gene expression groups, respectively. The colored stars on IPP matrices indicate various patient stratification cases corresponding to Kaplan-Meier plots in the figure. (B–D) Kaplan-Meier plots showing survival probabilities of patients in the cases corresponding to the colored stars in IPP matrix (A). (F–H) Kaplan-Meier plots showing survival probabilities of patients in the cases corresponding to the colored stars in IPP matrix (E). (J–L) Kaplan-Meier plots showing survival probabilities of patients in the cases corresponding to the colored stars in IPP matrix (I). (N–P) Kaplan-Meier plots showing survival probabilities of patients in the cases corresponding to the colored stars in IPP matrix (M). The p-values and hazard ratios were calculated for the log-rank test. Gene expression thresholds in B, F, J, and N are the average gene expression levels for all total subjects in cases A, E, I, and M, respectively. All cases for Kaplan-Meier plots except N showed insignificant differences of survival probabilities between two groups by the log-rank test.

Supplementary Figure 2. Histogram showing the IPP score distributions among 21 breast cancer datasets.

X-axis: bin center of IPP scores, Y-axis: the number of genes included within each bin. Statistics are provided in Supplementary Table 2.

Supplementary Figure 3. Histograms of z-score distributions obtained using the log-rank test with the average value taken as the gene expression level threshold.

X-axis: bin center of z-scores, Y-axis: the number of genes included within each bin. Statistics are provided in Supplementary Table 3.

Supplementary Figure 4. Histograms of z-score distributions obtained using the log-rank test with the median value taken as the gene expression level threshold.

X-axis: bin center of z-scores, Y-axis: the number of genes included within each bin. Statistics are provided in

Supplementary Table 4.

Supplementary Figure 5. Box and whisker plots of the distributions of IPP scores, and z-scores of the log-rank test, among datasets according to average and median gene expression level thresholds.

(A) Box and whisker plots of IPP score distributions among 21 breast cancer datasets. (B) Box and whisker plots of log-rank test z-score distributions among 21 breast cancer datasets using the average value as the gene expression threshold. (C) Box and whisker plots of z-score distributions among 21 breast cancer datasets using the median value as the gene expression threshold. Datasets in (A), (B), and (C) are in the following order: E-MTAB-365, E-MTAB-748, E-TABM-158, GSE11121, GSE12093, GSE16446, GSE17705, GSE17907, GSE19615, GSE2034, GSE20685, GSE2603, GSE45255, GSE5327, GSE58644, GSE58812, GSE6532_KI, GSE6532_OXF (GPL96), GSE6532_OXF (GPL570), GSE7390, and GSE9195. Lower and upper whiskers represent the minimum and maximum of score distributions, respectively. Upper and lower borders of the box plots represent the 75th and 25th percentiles, respectively.

Supplementary Figure 6. IPP identified prognostic genes more robustly than the conventional log-rank test using the median value as the gene expression level threshold.

(A) The number of prognostic genes shared by independent datasets, as identified by IPP and the log-rank test using the median value as the threshold for gene expression level. A gene is considered prognostic if its absolute score (IPP score in IPP or z-score in the log-rank test) is within the top 5 % of genes; the same number of genes was analyzed using IPP and the log-rank test to ensure a fair comparison of the genes shared among datasets. Prognostic genes shared among five or more datasets are represented by bar graphs. (B) Venn diagram (left panel) showing the number of genes identified only by IPP, only by the log-rank test, and by both methods. No functional groups were found for genes identified only by the log-rank test using Reactome pathway enrichment analysis (right panel). (C) The IPP scores represented more reliable when they were compared to the scores from subsamples versus the log-rank test by bootstrapping. For each subsample on the x-axis, 1,000 repeated bootstrapping iterations were performed and the average values were plotted. For the log-rank test, the z-score was used instead of the p-value. Error bars, mean ± SD. (D) Outcome relation (adverse or favorable) of the genes were determined more consistently by IPP than by the log-rank test. The numbers on bar graphs are the numbers

of genes with identical outcome relation (adverse or favorable) among 17 or more datasets. (E) The IPP scores represented more reliable when they were compared to the scores from subsamples versus log-rank test, using average (left panel) and median (right panel) values as expression level thresholds by subsampling. For each subsample on the x-axis, 1,000 repeated subsampling iterations were performed, and the average values were plotted. For the log-rank test, the z-score was used instead of the p-value. Error bars, mean ± SD. The cyan, purple, and green colors indicate the results from IPP, and log-rank tests using the average and median values as thresholds, respectively. "Log-rank test: Avg" and "Log-rank test: Med" indicate the results obtained from log-rank tests using the average and median values as thresholds, respectively.

Supplementary Figure 7. Robustness comparison between IPP and conventional log-rank tests using average or median values as gene expression level thresholds, through sampling of Pearson correlation and outcome relation consistency.

(A) Comparison of Pearson's r between the scores from all samples and subsamples obtained by bootstrapping (upper panel) and subsampling (lower panel) with the IPP and log-rank tests using average (left in each dataset) and median values (right in each dataset) as gene expression level thresholds. Error bars, mean ± SD. (B) Distribution of genes in each adverse-favorable pair. Left, comparison of the results between IPP and the log-rank test using the average value as the gene expression level threshold. Right, comparison of results between IPP and the log-rank test using the median value as the gene expression level threshold. X-axis: number of adverse-favorable pairs. Y-axis: the number of genes included in each case. "Log-rank test: Avg" and "Log-rank test: Med" indicate results obtained from log-rank tests using the average and median values as thresholds, respectively.

Supplementary Figure 8. IPP score distributions for arbitrary, randomly constructed patient samples.

X-axis of histogram: bin center of IPP scores, Y-axis of histogram: the number of genes included in each bin. Box and whisker plots show IPP score distributions for all randomly constructed patient samples. Lower and upper whiskers represent the minimum and maximum values of each score distribution, respectively. Upper and lower borders of the box plots represent the 75th and 25th percentiles, respectively. The statistics are summarized in Supplementary Table 5.

Supplementary Figure 9. IPP score distributions by breast cancer subtype: luminal (ER-positive/PR-positive), HER2-enriched (ER-negative/PR-negative/HER2-positive), and triple-negative (ER-negative/PR-negative/HER2-negative).

(A) IPP score distributions for luminal breast cancer. (B) IPP score distributions for triple-negative breast cancer. (C) IPP score distributions for HER2-enriched breast cancer. (D) Box and whisker plots show the distributions of the IPP scores according to liptak's weighted method. X-axis of histogram: bin center of IPP scores, Y-axis of histogram: the number of genes included in each bin. Lower and upper whiskers represent the minimum and maximum values of each score distribution, respectively. Upper and lower borders of the box plots represent the 75th and 25th percentiles, respectively. Statistical results are provided in Supplementary Tables 6-8.

Supplementary Figure 10. Functional groups of prognostic genes in HER2-enriched (ER-negative/PR-negative/HER2-positive) breast cancer.

(A) In total, 62.7 % and 37.3 % of all prognostic genes (n = 577) were adverse and favorable prognostic genes for HER2-enriched breast cancer, respectively. Among all prognostic genes, 37.7 % and 25.6 % of adverse and favorable prognostic genes, respectively, were assigned to functional groups by Reactome pathway enrichment analysis (upper panel). Representative functional groups of adverse (red) and favorable (blue) prognostic genes in HER2-enriched breast cancer (lower panel). X-axis: common logarithm of the FDR. (B) Among all prognostic genes (n = 577) for HER2-enriched breast cancer, 25.0 % and 11.7 % of adverse and favorable prognostic genes, respectively, were not assigned to any functional group by Reactome pathway enrichment analysis (upper panel). Kaplan-Meier curves show prognostic differences in DFS (days) and OS (days) according to the CNA state (amplification, neutral, or deletion) of C21orf91 and METTL17, which are favorable and adverse prognostic genes, respectively, unassigned to a functional group by Reactome pathway enrichment analysis (lower panel). Bar colors indicate the outcome relation (adverse or favorable) and enriched or non-enriched prognostic genes. The colors of the Kaplan-Meier curves indicate the CNA states (purple: amplification, black: neutral, and green: deletion). Each n on Kaplan-Meier curves indicates the number of patients included in that CNA state.

Supplementary Figure 11. Protein-Protein Interaction (PPI) network pathway of luminal (ER-positive/PR-positive) breast cancer.

In total, 50.2 % (n = 234) and 14.3 % (n = 13) of the adverse (upper panel) and favorable (lower panel) prognostic genes, respectively, had at least one PPI with each other and thus organize the pathway networks. Percentages indicate the ratio between adverse and favorable prognostic genes.

Supplementary Figure 12. PPI network pathway of HER2-enriched (ER-negative/PR-negative/HER2-positive) breast cancer.

In total, 30.1 % (n = 105) and 22.1 % (n = 44) of the adverse (upper panel) and favorable (lower panel) prognostic genes, respectively, had at least one PPI with each other and thus organize the pathway networks. Percentages indicate the gene number ratio based on adverse and favorable prognostic genes.

Supplementary Figure 13. PPI network pathway of triple-negative (ER-negative/PR-negative/HER2-negative) breast cancer.

In total, 27.0 % (n = 98) and 51.0 % (n = 99) of the adverse (upper panel) and favorable (lower panel) prognostic genes, respectively, had at least one PPI with each other and thus organize the pathway networks. Percentages indicate the ratio between adverse and favorable prognostic genes.

Supplementary Figure 14. Prognostic significance of mutations of functionally unknown prognostic genes identified by IPP.

Kaplan-Meier curves of six representative prognostic genes (DONSON, MKI67, FAM171A1, TENM4, C16orf45, and RABEP2), not assigned to any functional group by Reactome pathway enrichment analysis, showing the difference in OS (days) between patients with and without mutations. All patient samples within the BRCA-US data of the ICGC database were used in this calculation, because there are no data to allow classification into luminal, her2-enriched, and triple-negative types. The colors of the Kaplan-Meier curves indicate different mutation states (purple: mutation, black: non-mutation). Each n on the Kaplan-Meier curves indicates the number of patients included within that different mutation state.

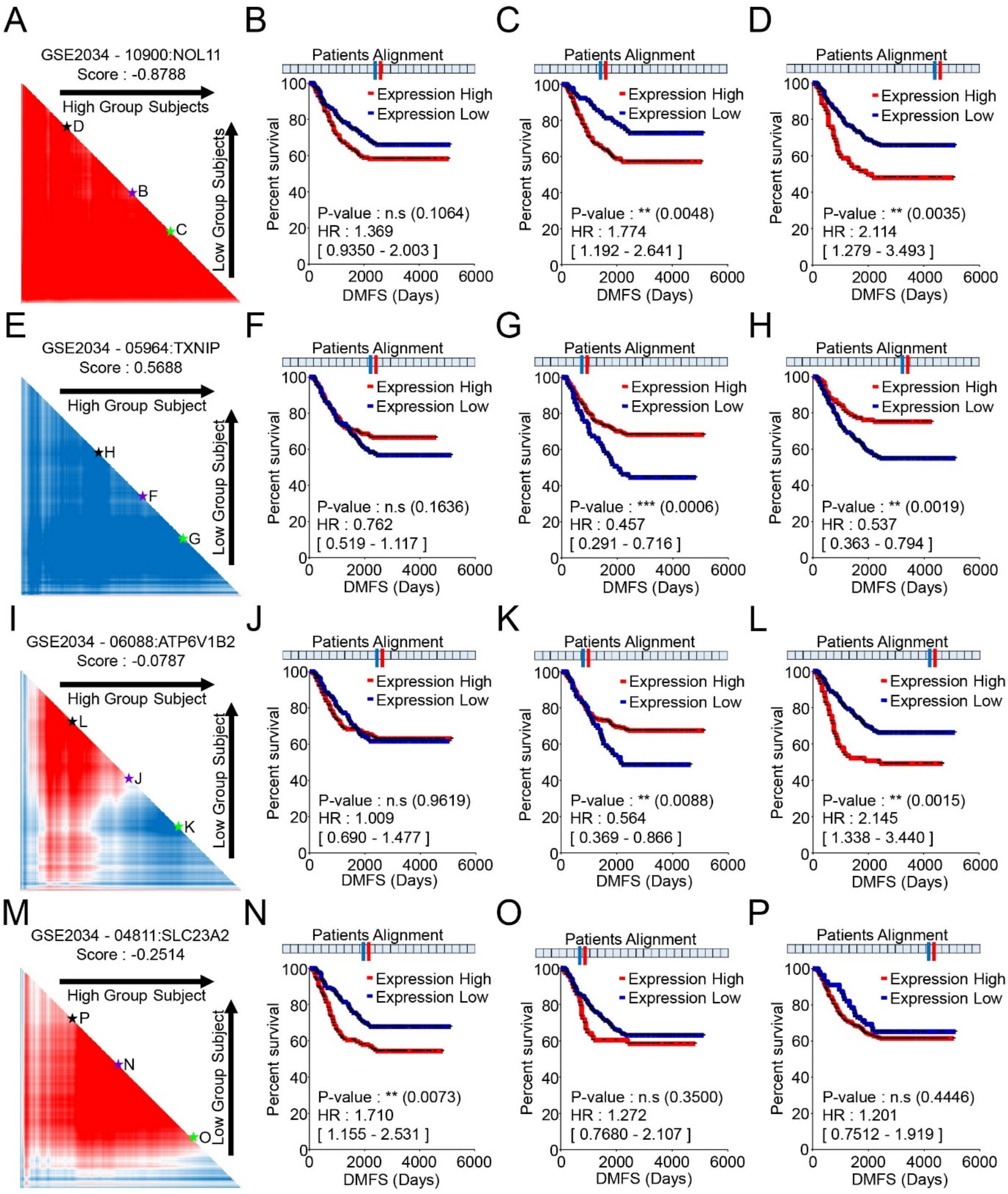

A
GSE2034 - 10900:NOL11
Score : -0.8788
High Group Subjects
Low Group Subjects
B
C
D
Patients Alignment
Expression High
Expression Low
Percent survival
DMFS (Days)
P-value : n.s (0.1064)
HR : 1.369
[0.9350 - 2.003]
P-value : ** (0.0048)
HR : 1.774
[1.192 - 2.641]
P-value : ** (0.0035)
HR : 2.114
[1.279 - 3.493]
E
GSE2034 - 05964:TXNIP
Score : 0.5688
High Group Subject
Low Group Subject
F
G
H
P-value : n.s (0.1636)
HR : 0.762
[0.519 - 1.117]
P-value : *** (0.0006)
HR : 0.457
[0.291 - 0.716]
P-value : ** (0.0019)
HR : 0.537
[0.363 - 0.794]
I
GSE2034 - 06088:ATP6V1B2
Score : -0.0787
J
K
L
P-value : n.s (0.9619)
HR : 1.009
[0.690 - 1.477]
P-value : ** (0.0088)
HR : 0.564
[0.369 - 0.866]
P-value : ** (0.0015)
HR : 2.145
[1.338 - 3.440]
M
GSE2034 - 04811:SLC23A2
Score : -0.2514
N
O
P
P-value : ** (0.0073)
HR : 1.710
[1.155 - 2.531]
P-value : n.s (0.3500)
HR : 1.272
[0.7680 - 2.107]
P-value : n.s (0.4446)
HR : 1.201
[0.7512 - 1.919]

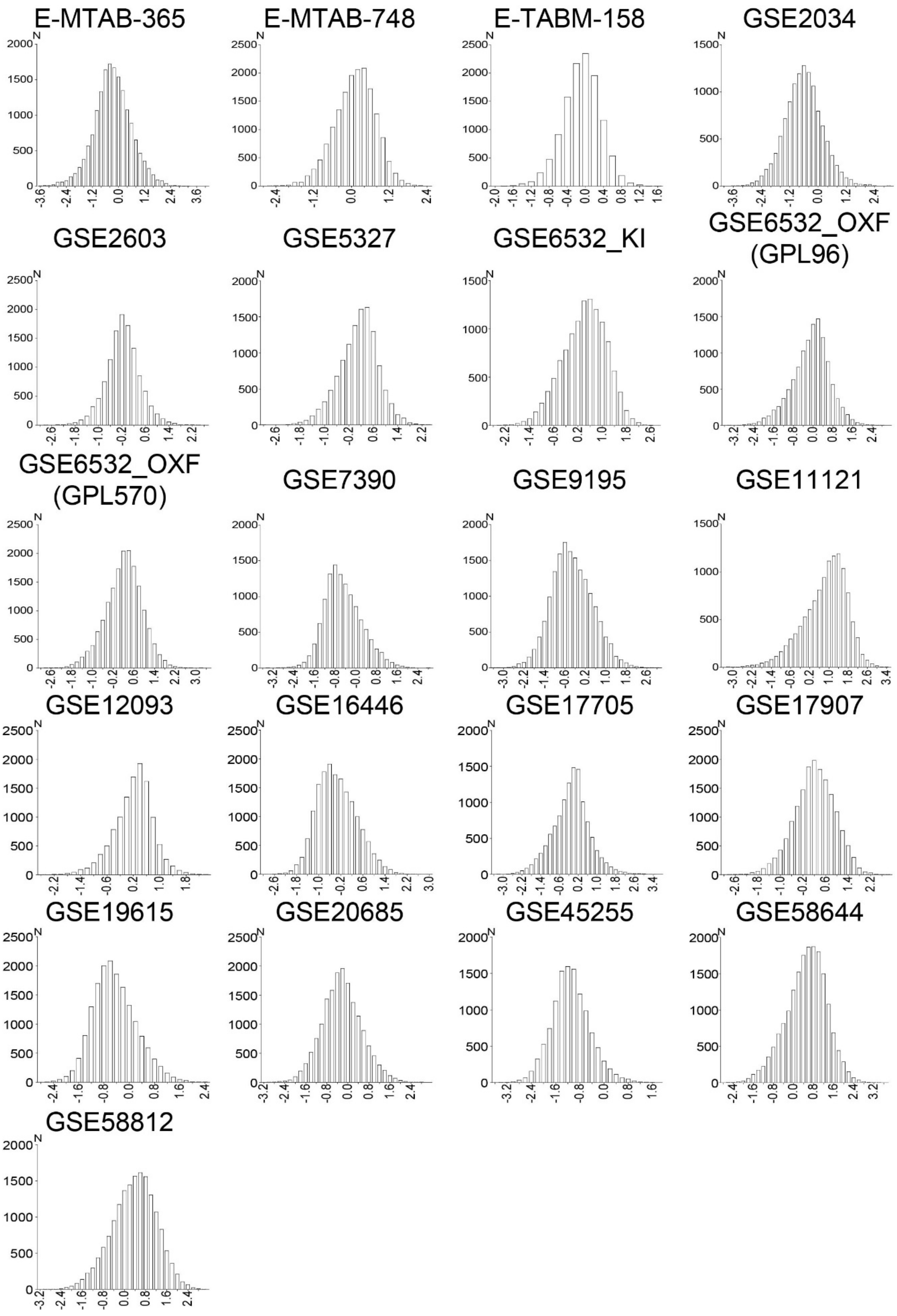
E-MTAB-365
E-MTAB-748
E-TABM-158
GSE2034
GSE2603
GSE5327
GSE6532_KI
GSE6532_OXF (GPL96)
GSE6532_OXF (GPL570)
GSE7390
GSE9195
GSE11121
GSE12093
GSE16446
GSE17705
GSE17907
GSE19615
GSE20685
GSE45255
GSE58644
GSE58812
N

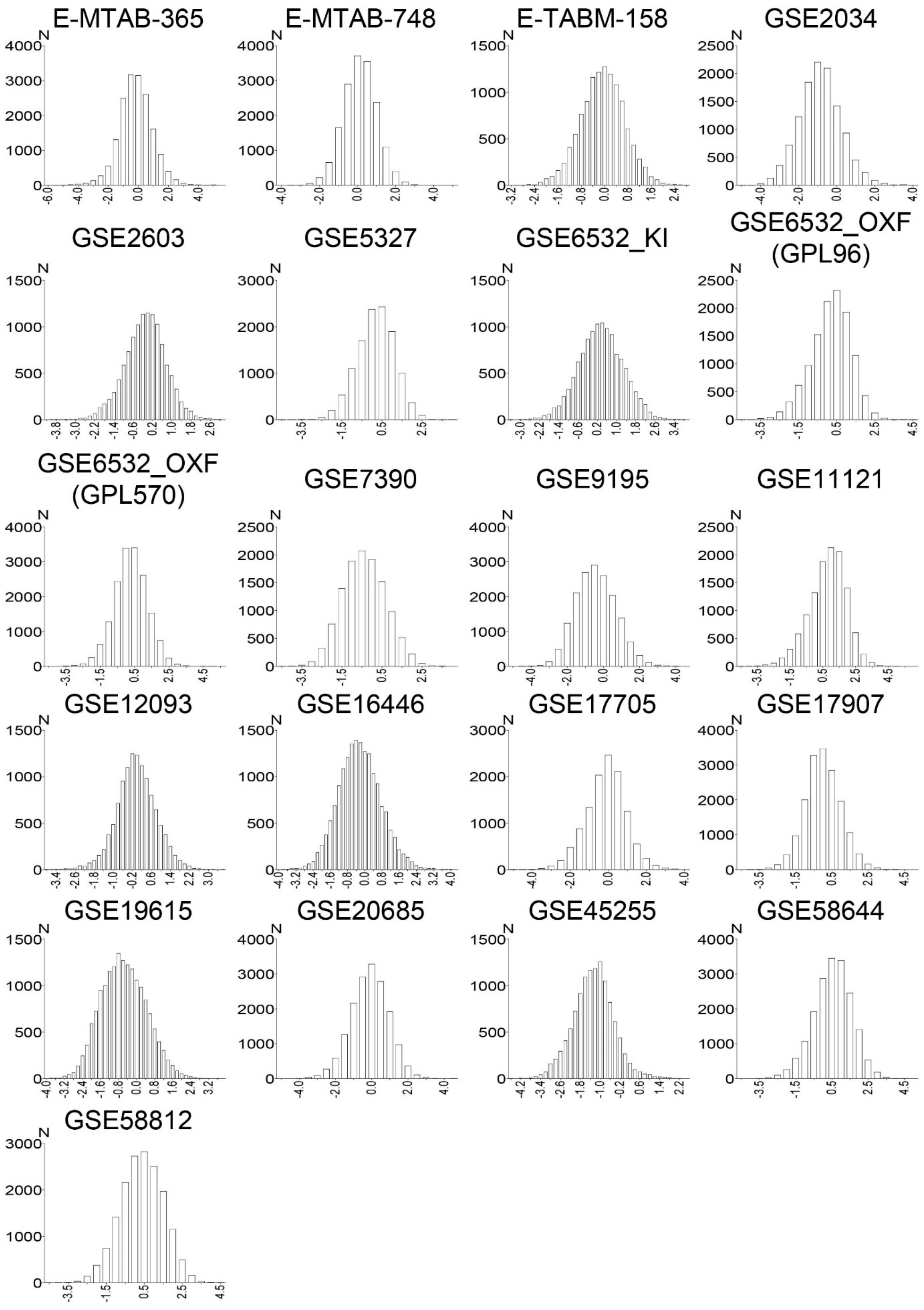
E-MTAB-365
E-MTAB-748
E-TABM-158
GSE2034
GSE2603
GSE5327
GSE6532_KI
GSE6532_OXF (GPL96)
GSE6532_OXF (GPL570)
GSE7390
GSE9195
GSE11121
GSE12093
GSE16446
GSE17705
GSE17907
GSE19615
GSE20685
GSE45255
GSE58644
GSE58812
N

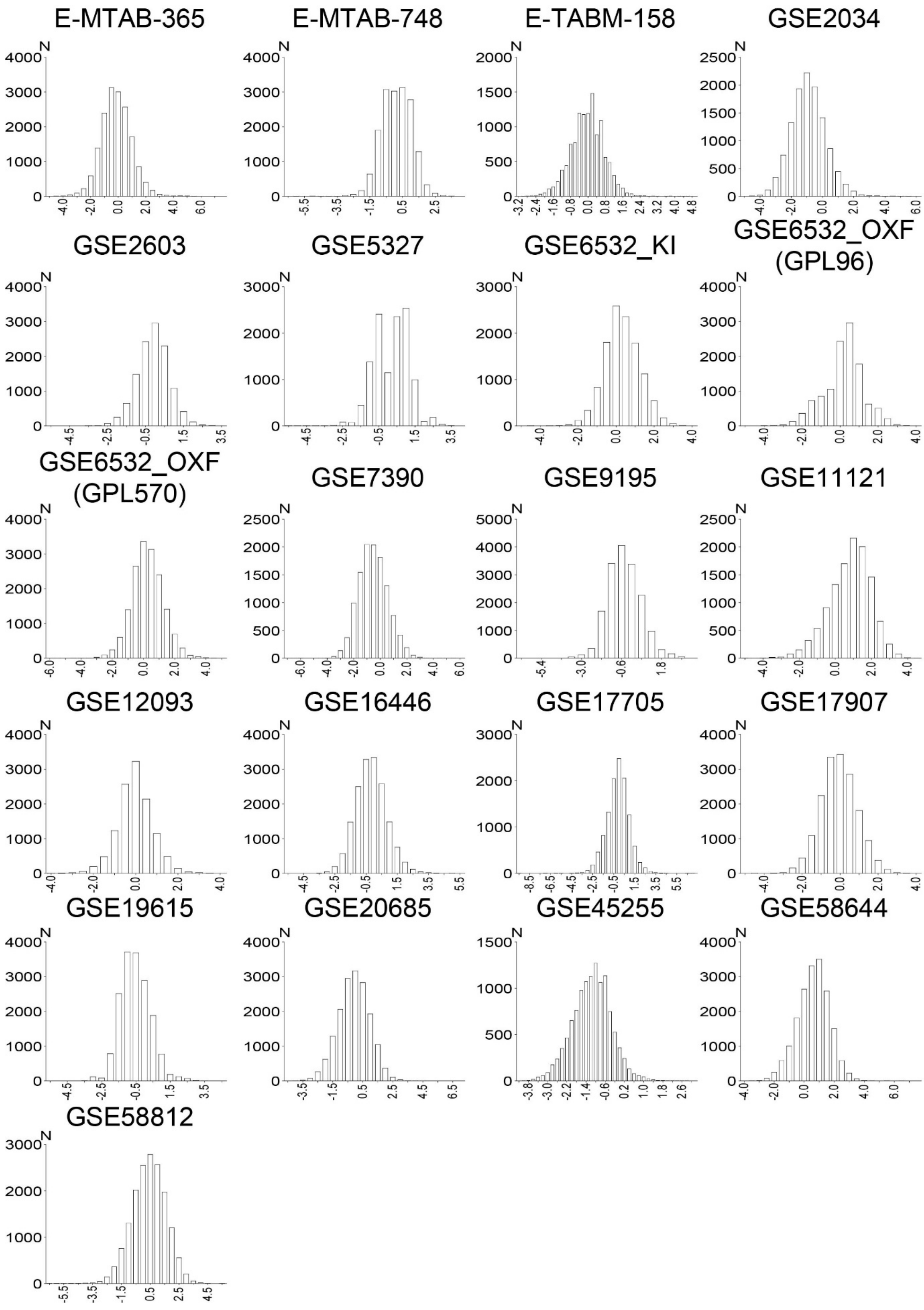
E-MTAB-365
E-MTAB-748
E-TABM-158
GSE2034
GSE2603
GSE5327
GSE6532_KI
GSE6532_OXF (GPL96)
GSE6532_OXF (GPL570)
GSE7390
GSE9195
GSE11121
GSE12093
GSE16446
GSE17705
GSE17907
GSE19615
GSE20685
GSE45255
GSE58644
GSE58812

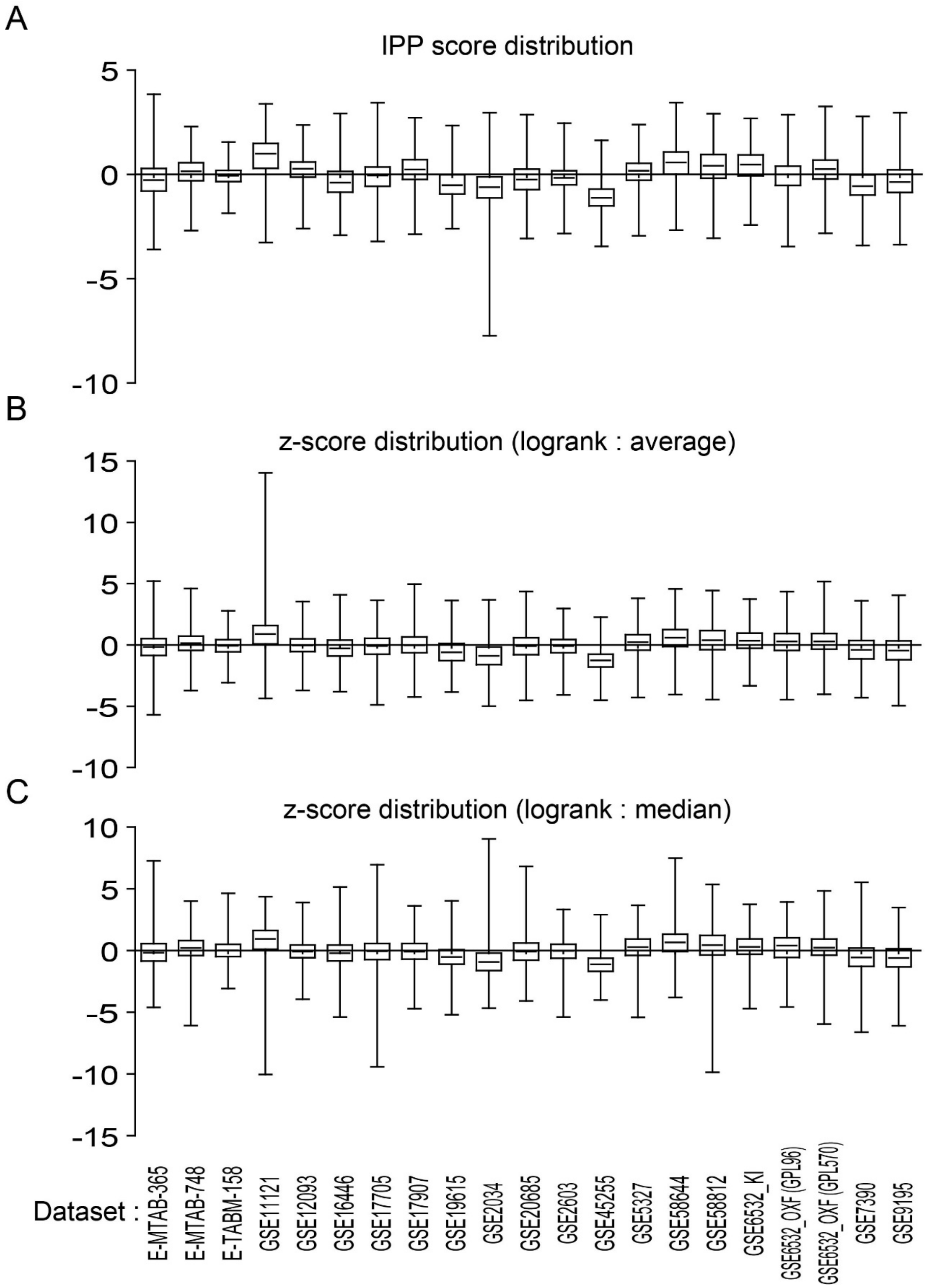

A
IPP score distribution
5
0
-5
-10
B
z-score distribution (logrank : average)
15
10
5
0
-5
-10
C
z-score distribution (logrank : median)
10
5
0
-5
-10
-15
Dataset :
E-MTAB-365
E-MTAB-748
E-TABM-158
GSE11121
GSE12093
GSE16446
GSE17705
GSE17907
GSE19615
GSE2034
GSE20685
GSE2603
GSE45255
GSE5327
GSE58644
GSE58812
GSE6532_KI
GSE6532_OXF (GPL96)
GSE6532_OXF (GPL570)
GSE7390
GSE9195

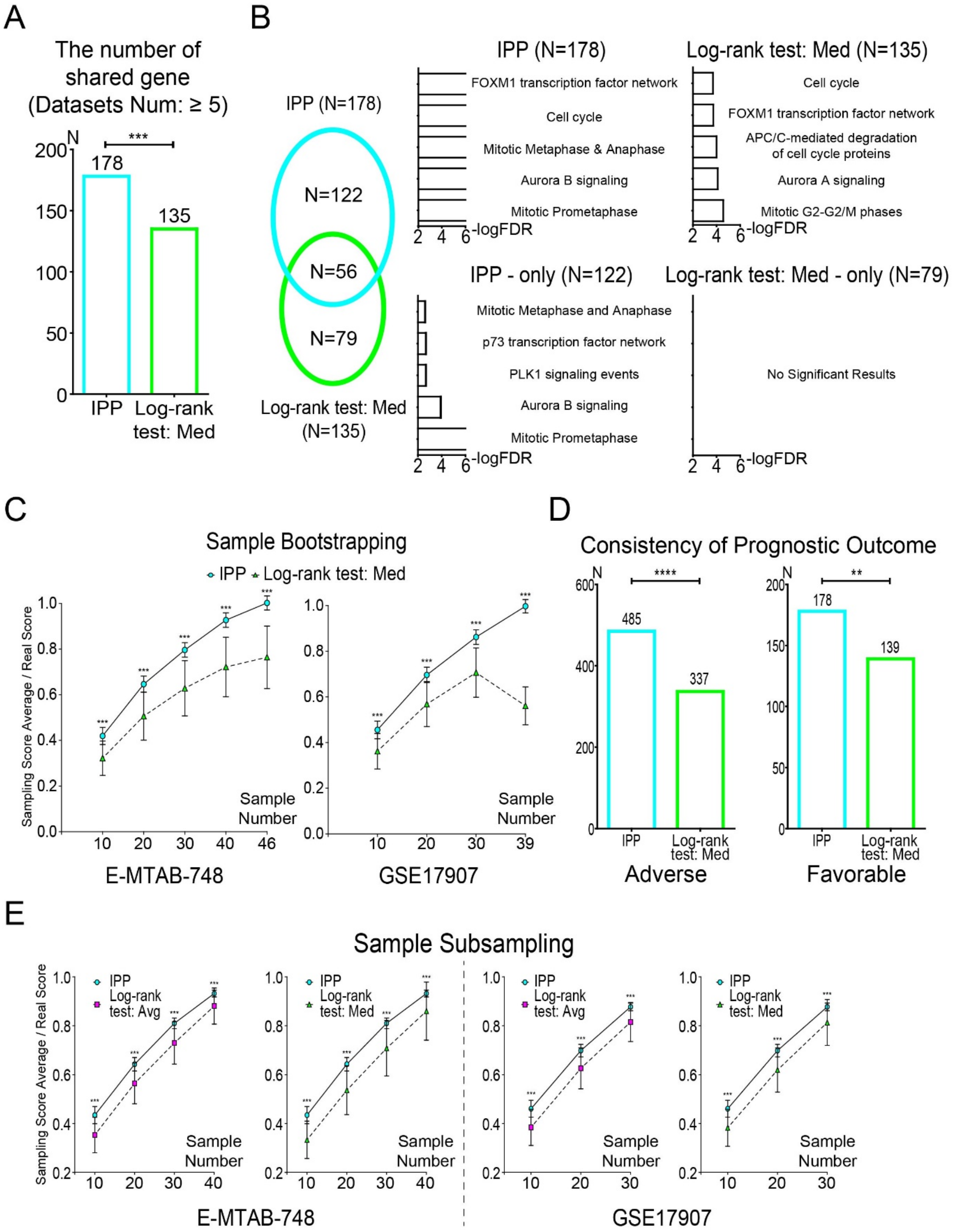
A
The number of shared gene (Datasets Num: ≥ 5)
N
***
178
135
IPP
Log-rank test: Med
B
IPP (N=178)
N=122
N=56
N=79
Log-rank test: Med (N=135)
IPP (N=178)
FOXM1 transcription factor network
Cell cycle
Mitotic Metaphase & Anaphase
Aurora B signaling
Mitotic Prometaphase
-logFDR
Log-rank test: Med (N=135)
Cell cycle
FOXM1 transcription factor network
APC/C-mediated degradation of cell cycle proteins
Aurora A signaling
Mitotic G2-G2/M phases
IPP - only (N=122)
Mitotic Metaphase and Anaphase
p73 transcription factor network
PLK1 signaling events
Aurora B signaling
Mitotic Prometaphase
Log-rank test: Med - only (N=79)
No Significant Results
C
Sample Bootstrapping
IPP
Log-rank test: Med
Sampling Score Average / Real Score
Sample Number
E-MTAB-748
GSE17907
D
Consistency of Prognostic Outcome
N
****
485
337
**
178
139
IPP
Log-rank test: Med
Adverse
Favorable
E
Sample Subsampling
IPP
Log-rank test: Avg
Log-rank test: Med
Sampling Score Average / Real Score
Sample Number
E-MTAB-748
GSE17907

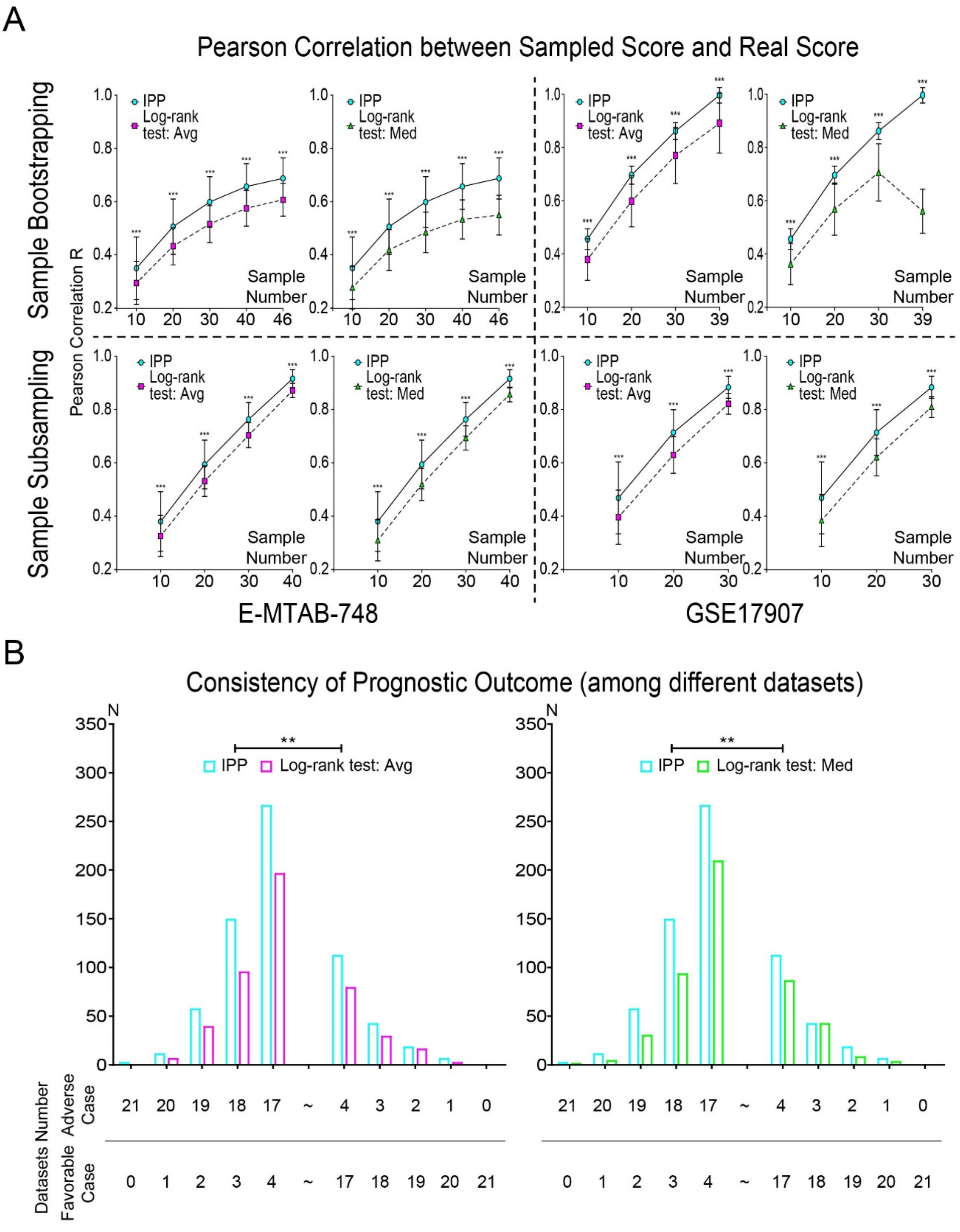
A
Pearson Correlation between Sampled Score and Real Score
Sample Bootstrapping
Sample Subsampling
Pearson Correlation R
IPP
Log-rank test: Avg
Log-rank test: Med
Sample Number
E-MTAB-748
GSE17907
B
Consistency of Prognostic Outcome (among different datasets)
N
IPP
Log-rank test: Avg
Log-rank test: Med
Datasets Number
Adverse Case
Favorable Case

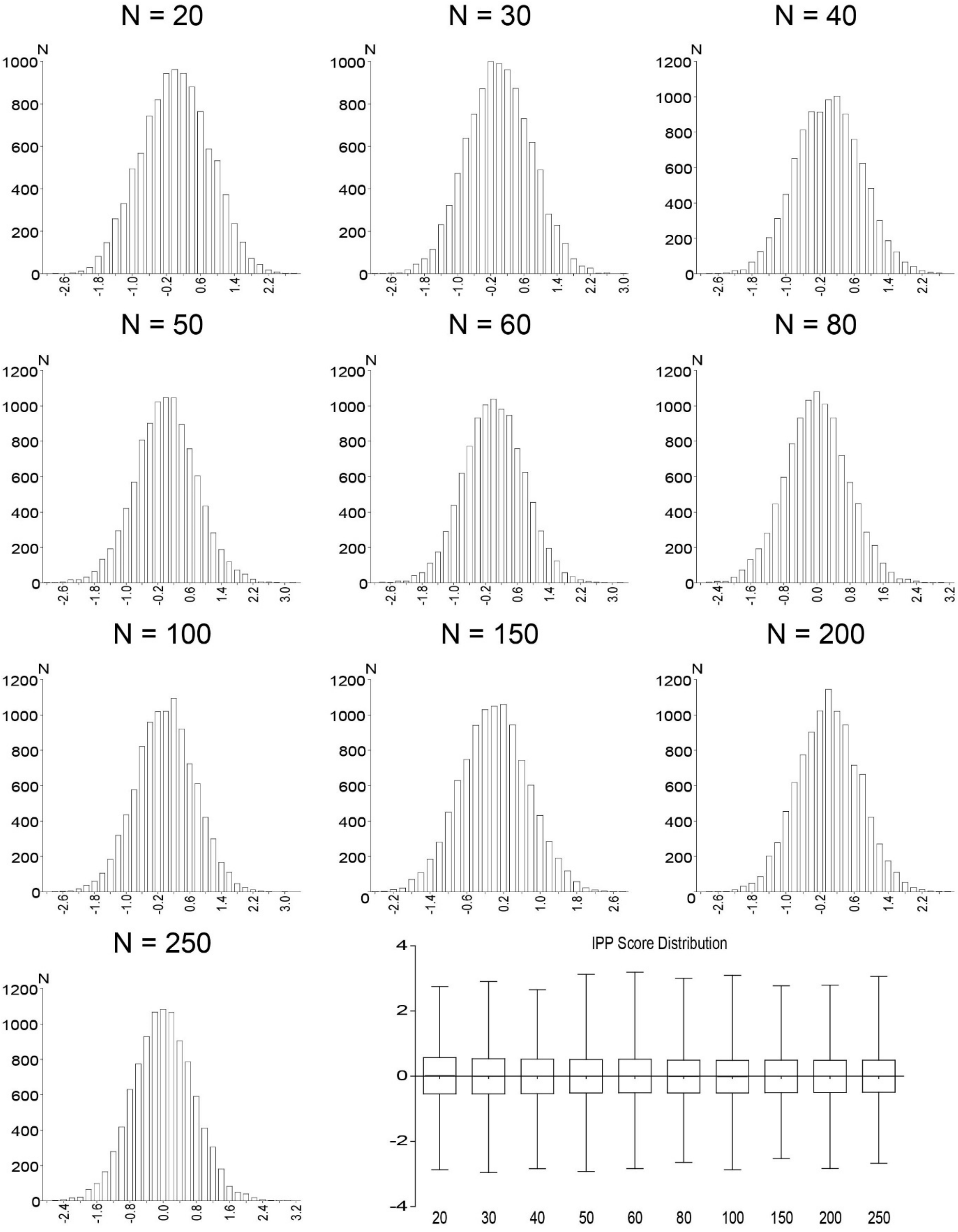
N = 20
N = 30
N = 40
N = 50
N = 60
N = 80
N = 100
N = 150
N = 200
N = 250
N
0
200
400
600
800
1000
1200
-2.6
-1.8
-1.0
-0.2
0.6
1.4
2.2
3.0
-2.4
-1.6
-0.8
0.0
0.8
1.6
2.4
3.2
-2.2
-1.4
-0.6
0.2
1.0
1.8
2.6
IPP Score Distribution
4
2
0
-2
-4
20
30
40
50
60
80
100
150
200
250

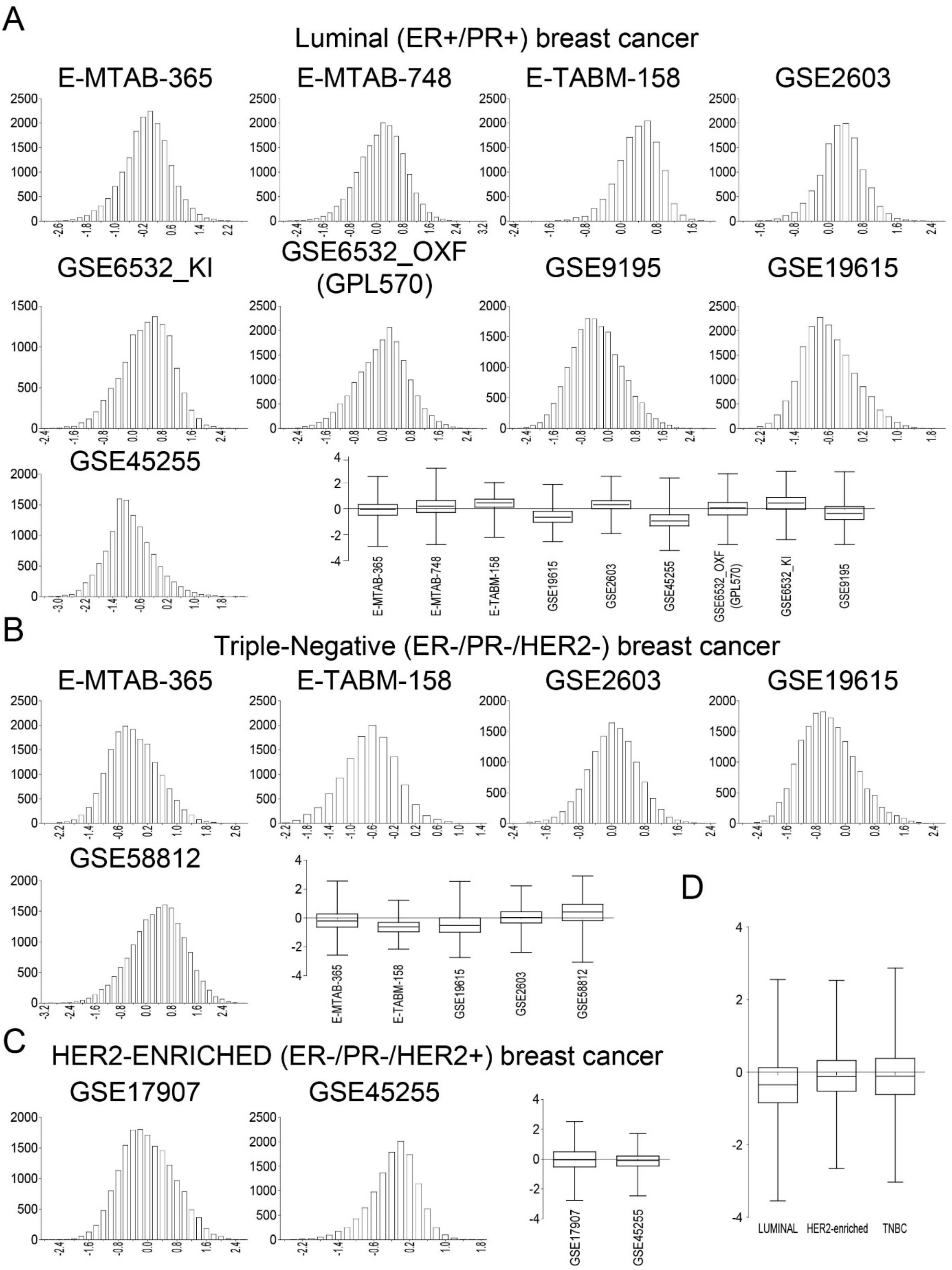
A
Luminal (ER+/PR+) breast cancer
E-MTAB-365
E-MTAB-748
E-TABM-158
GSE2603
GSE6532_KI
GSE6532_OXF (GPL570)
GSE9195
GSE19615
GSE45255
B
Triple-Negative (ER-/PR-/HER2-) breast cancer
E-MTAB-365
E-TABM-158
GSE2603
GSE19615
GSE58812
C
HER2-ENRICHED (ER-/PR-/HER2+) breast cancer
GSE17907
GSE45255
D
LUMINAL
HER2-enriched
TNBC

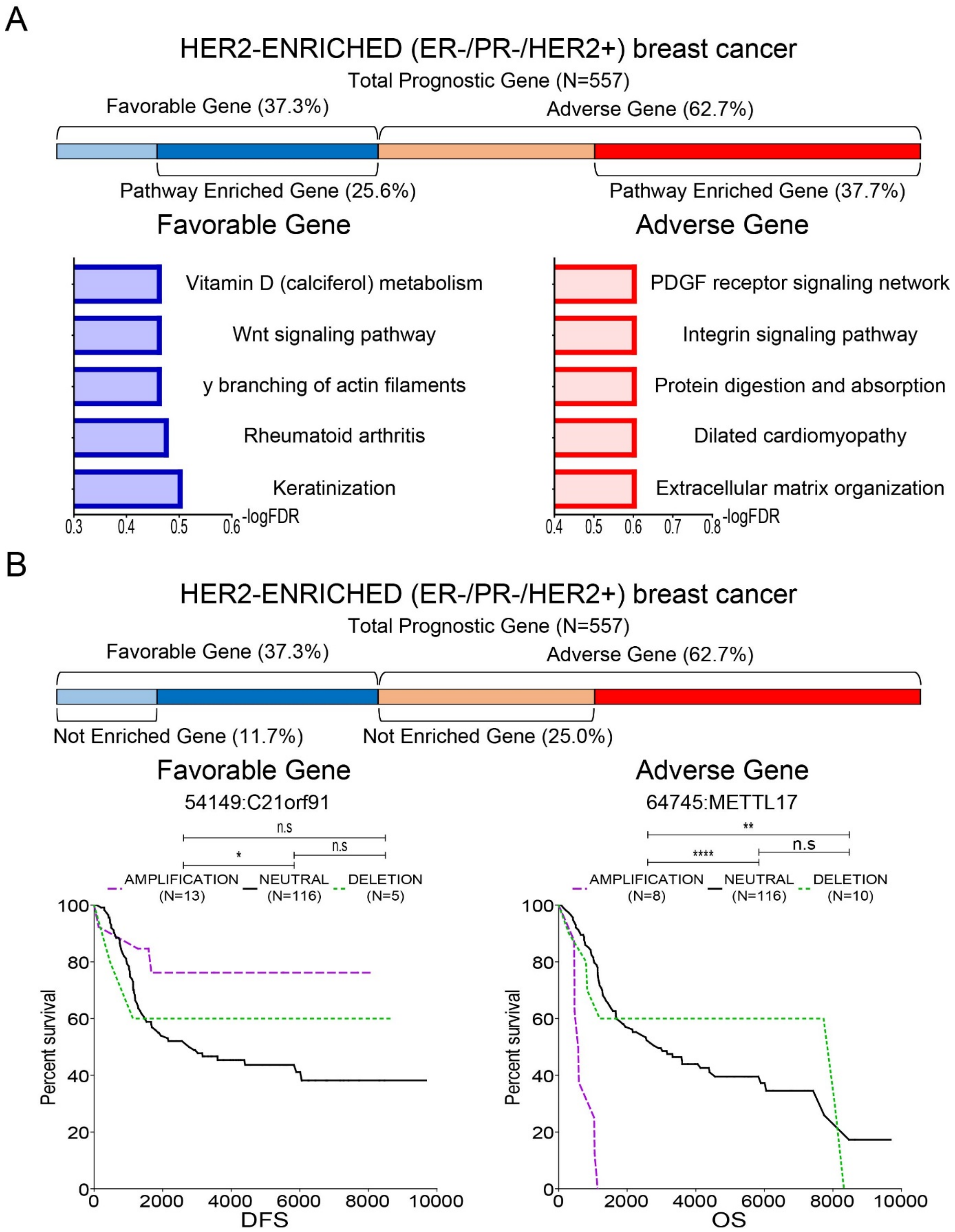

A
HER2-ENRICHED (ER-/PR-/HER2+) breast cancer
Total Prognostic Gene (N=557)
Favorable Gene (37.3%)
Adverse Gene (62.7%)
Pathway Enriched Gene (25.6%)
Pathway Enriched Gene (37.7%)
Favorable Gene
Adverse Gene
Vitamin D (calciferol) metabolism
Wnt signaling pathway
y branching of actin filaments
Rheumatoid arthritis
Keratinization
PDGF receptor signaling network
Integrin signaling pathway
Protein digestion and absorption
Dilated cardiomyopathy
Extracellular matrix organization
-logFDR
B
HER2-ENRICHED (ER-/PR-/HER2+) breast cancer
Total Prognostic Gene (N=557)
Favorable Gene (37.3%)
Adverse Gene (62.7%)
Not Enriched Gene (11.7%)
Not Enriched Gene (25.0%)
Favorable Gene
54149:C21orf91
Adverse Gene
64745:METTL17
AMPLIFICATION (N=13)
NEUTRAL (N=116)
DELETION (N=5)
AMPLIFICATION (N=8)
NEUTRAL (N=116)
DELETION (N=10)
Percent survival
DFS
OS

Luminal (ER+/PR+) breast cancer

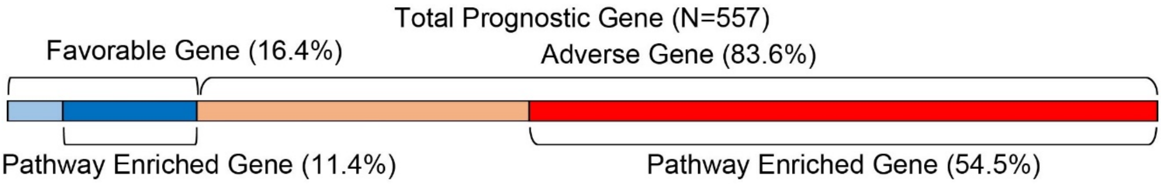

Reactome Pathway - Adverse Gene (N=234, 50.2%)

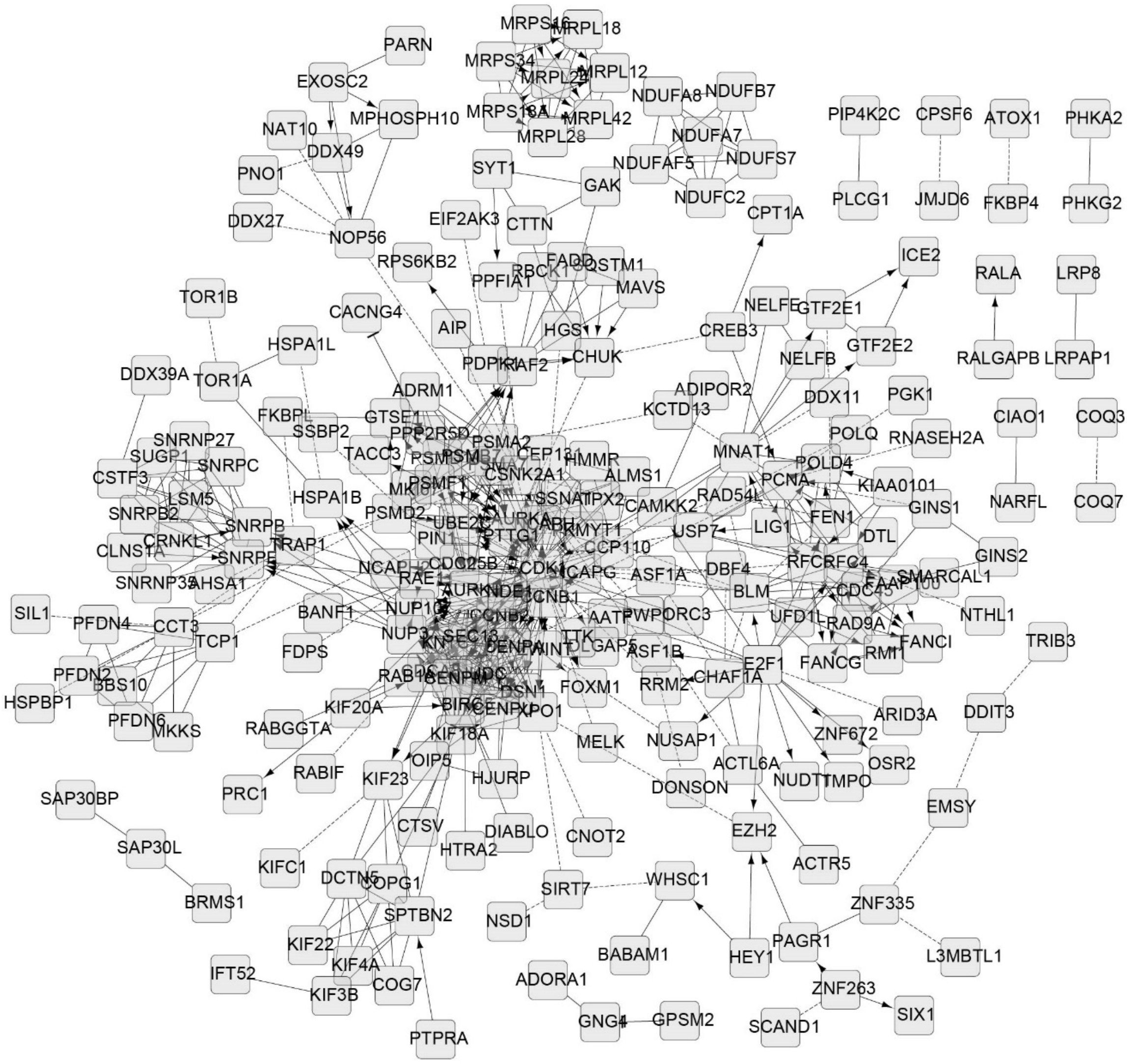

Reactome Pathway - Favorable Gene (N=13, 14.3%)

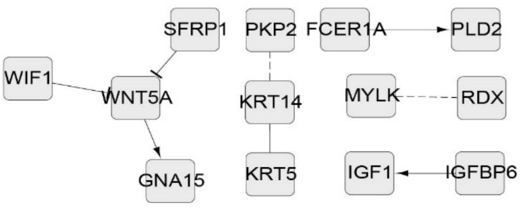

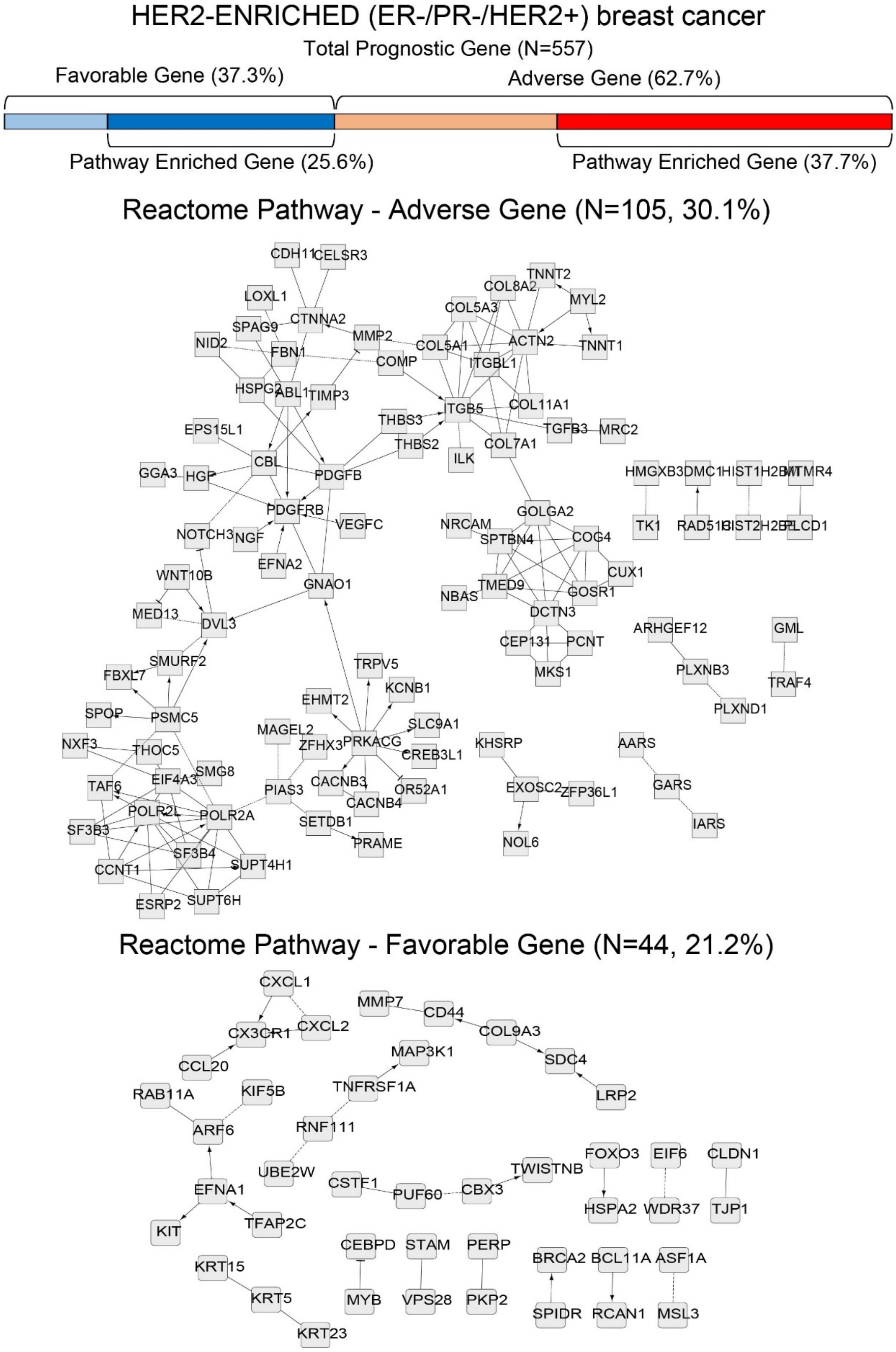
HER2-ENRICHED (ER-/PR-/HER2+) breast cancer
Total Prognostic Gene (N=557)
Favorable Gene (37.3%)
Adverse Gene (62.7%)
Pathway Enriched Gene (25.6%)
Pathway Enriched Gene (37.7%)
Reactome Pathway - Adverse Gene (N=105, 30.1%)
CDH11
CELSR3
LOXL1
CTNNA2
SPAG9
NID2
FBN1
MMP2
COMP
HSPG2
ABL1
TIMP3
TNNT2
COL8A2
MYL2
COL5A3
ACTN2
COL5A1
TNNT1
ITGBL1
ITGB5
COL11A1
THBS3
TGFB3
MRC2
THBS2
COL7A1
ILK
EPS15L1
CBL
GGA3
HGF
PDGFB
PDGFRB
VEGFC
NOTCH3
NGF
EFNA2
GNAO1
WNT10B
MED13
DVL3
SMURF2
FBXL7
SPOP
PSMC5
NXF3
THOC5
EIF4A3
SMG8
TAF6
POLR2L
POLR2A
SF3B3
SF3B4
CCNT1
SUPT4H1
ESRP2
SUPT6H
TRPV5
KCNB1
EHMT2
SLC9A1
MAGEL2
ZFHX3
PRKACG
CREB3L1
CACNB3
OR52A1
CACNB4
PIAS3
SETDB1
PRAME
GOLGA2
NRCAM
SPTBN4
COG4
CUX1
TMED9
GOSR1
NBAS
DCTN3
CEP131
PCNT
MKS1
HMGXB3
DMC1
HIST1H2BM
MTMR4
TK1
RAD51B
HIST2H2BF
PLCD1
ARHGEF12
GML
PLXNB3
TRAF4
PLXND1
KHSRP
EXOSC2
ZFP36L1
NOL6
AARS
GARS
IARS
Reactome Pathway - Favorable Gene (N=44, 21.2%)
CXCL1
CX3CR1
CXCL2
CCL20
MMP7
CD44
COL9A3
MAP3K1
SDC4
LRP2
RAB11A
KIF5B
ARF6
TNFRSF1A
RNF111
UBE2W
EFNA1
KIT
TFAP2C
CSTF1
PUF60
CBX3
TWISTNB
FOXO3
HSPA2
EIF6
WDR37
CLDN1
TJP1
KRT15
KRT5
KRT23
CEBPD
MYB
STAM
VPS28
PERP
PKP2
BRCA2
SPIDR
BCL11A
RCAN1
ASF1A
MSL3

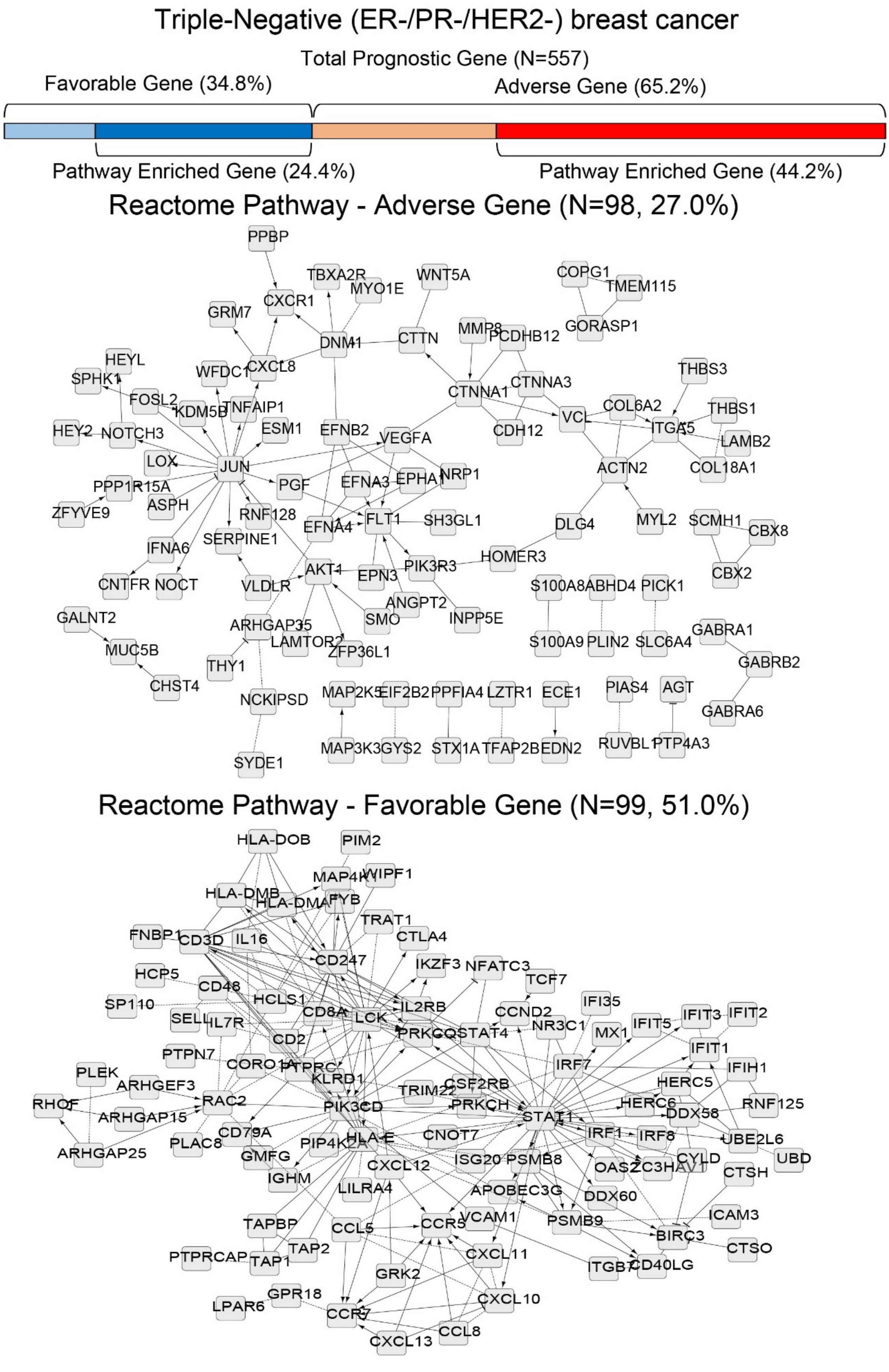
Triple-Negative (ER-/PR-/HER2-) breast cancer
Total Prognostic Gene (N=557)
Favorable Gene (34.8%)
Adverse Gene (65.2%)
Pathway Enriched Gene (24.4%)
Pathway Enriched Gene (44.2%)
Reactome Pathway - Adverse Gene (N=98, 27.0%)
PPBP
TBXA2R
MYO1E
WNT5A
COPG1
TMEM115
CXCR1
GRM7
DNM1
CTTN
MMP8
PCDHB12
GORASP1
HEYL
WFDC1
CXCL8
SPHK1
FOSL2
KDM5D
TNFAIP1
CTNNA3
CTNNA1
THBS3
COL6A2
THBS1
VCL
ITGA5
HEY2
NOTCH3
ESM1
EFNB2
VEGFA
CDH12
LAMB2
LOX
JUN
NRP1
ACTN2
COL18A1
PPP1R15A
PGF
EFNA3
EPHA1
ASPH
ZFYVE9
RNF128
FLT1
SH3GL1
DLG4
MYL2
SCMH1
CBX8
EFNA4
SERPINE1
IFNA6
HOMER3
CBX2
PIK3R3
AKT4
EPN3
CNTFR
NOCT
VLDLR
S100A8
ABHD4
PICK1
ANGPT2
GALNT2
SMO
INPP5E
ARHGAP35
GABRA1
MUC5B
LAMTOR2
S100A9
PLIN2
SLC6A4
ZFP36L1
GABRB2
THY1
CHST4
MAP2K5
EIF2B2
PPFIA4
LZTR1
ECE1
PIAS4
AGT
NCKIPSD
GABRA6
MAP3K3
GYS2
STX1A
TFAP2B
EDN2
RUVBL1
PTP4A3
SYDE1
Reactome Pathway - Favorable Gene (N=99, 51.0%)
HLA-DOB
PIM2
MAP4K1
WIPF1
HLA-DMB
HLA-DMA
FYB
TRAT1
FNBP1
CD3D
IL16
CTLA4
CD247
IKZF3
NFATC3
HCP5
TCF7
CD48
IFI35
SP110
HCLS1
IL2RB
CCND2
IFIT3
IFIT2
SELL
IL7R
CD8A
LCK
PRKCQ
STAT4
NR3C1
MX1
IFIT5
CD2
IFIT1
PTPN7
IRF7
CORO1A
PTPRC
IFIH1
PLEK
ARHGEF3
KLRD1
CSF2RB
HERC5
TRIM22
RHOF
RAC2
PIK3CD
PRKCH
HERC6
RNF125
STAT1
DDX58
ARHGAP15
CD79A
IRF1
IRF8
UBE2L6
PLAC8
PIP4K2A
HLA-E
CNOT7
ARHGAP25
GMFG
CYLD
UBD
ISG20
PSMB8
OASL
ZC3HAV1
CXCL12
IGHM
CTSH
LILRA4
APOBEC3G
DDX60
VCAM1
ICAM3
TAPBP
PSMB9
CCL5
CCR5
BIRC3
TAP2
CTSO
PTPRCAP
TAP1
CXCL11
ITGB7
CD40LG
GRK2
GPR18
CXCL10
LPAR6
CCR7
CCL8
CXCL13

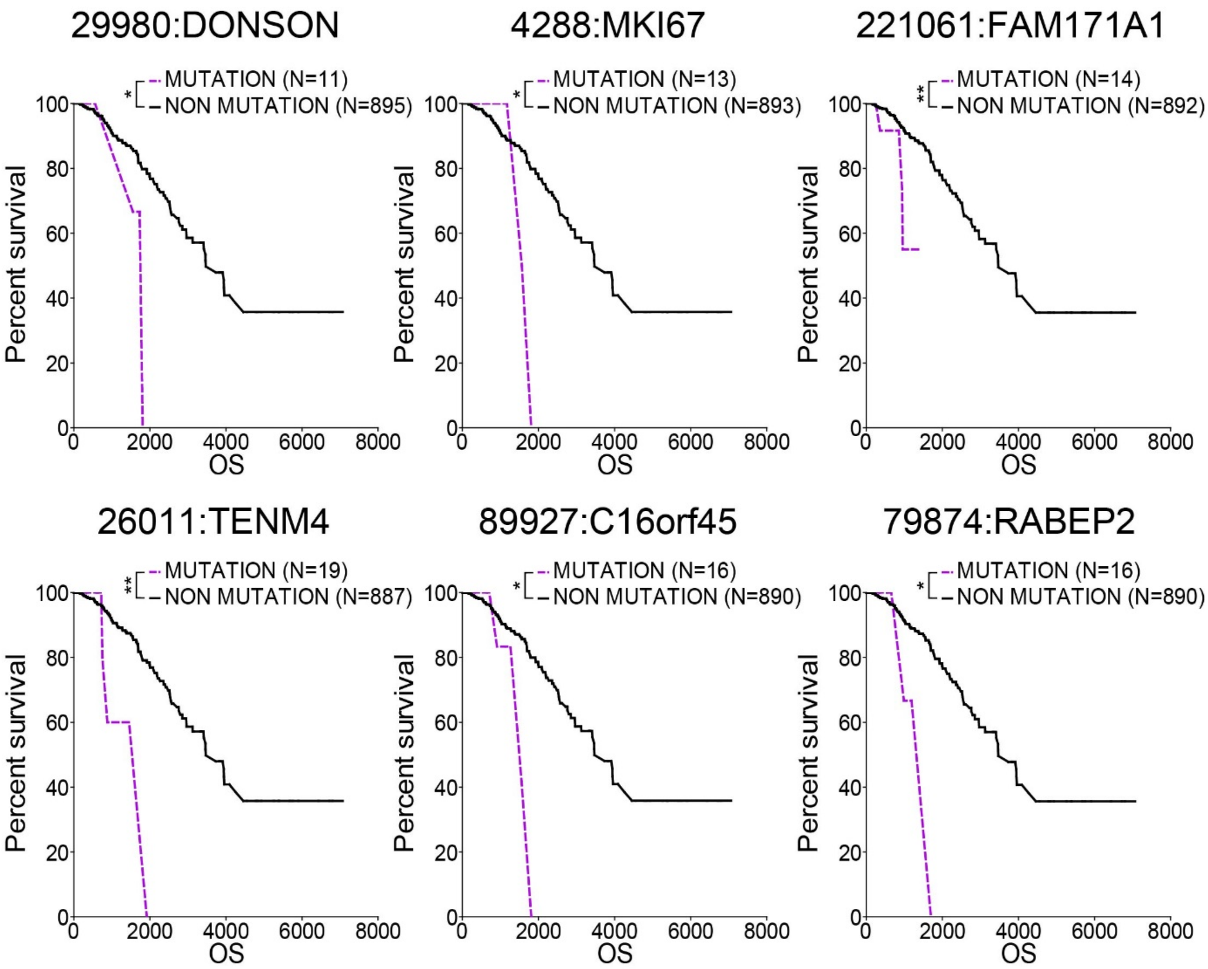
29980:DONSON
MUTATION (N=11)
NON MUTATION (N=895)
4288:MKI67
MUTATION (N=13)
NON MUTATION (N=893)
221061:FAM171A1
MUTATION (N=14)
NON MUTATION (N=892)
26011:TENM4
MUTATION (N=19)
NON MUTATION (N=887)
89927:C16orf45
MUTATION (N=16)
NON MUTATION (N=890)
79874:RABEP2
MUTATION (N=16)
NON MUTATION (N=890)
Percent survival
OS

Table S1. Breast Cancer Microarray Datasets

Microarray	Platform	Country	Used Patient Samples Number
GSE45255[47]	GPL96 [HG-U133A]	Singapore, Europe	136
GSE11121[48]	GPL96 [HG-U133A]	Germany	200
GSE2034[17]	GPL96 [HG-U133A]	USA	286
GSE58644[49]	GPL6244 [HuGene-1_0-st]	Canada	321
GSE58812[50]	GPL570 [HG-U133_Plus_2]	France	107
GSE6532[51]	GPL96 [HG-U133A]	UK	66
	GPL96 [HG-U133A]	Sweden	48
	GPL570 [HG-U133_Plus_2]	UK	87
E-MTAB-365[52]	GPL570 [HG-U133_Plus_2]	France	117
GSE7390[53]	GPL96 [HG-U133A]	Europe	198
GSE9195[54]	GPL570 [HG-U133_Plus_2]	UK	77
GSE17705[55]	GPL96 [HG-U133A]	USA, Belgium	298
GSE20685[56]	GPL570 [HG-U133_Plus_2]	Taiwan	83
GSE19615[57]	GPL570 [HG-U133_Plus_2]	USA	115
GSE17907[58]	GPL570 [HG-U133_Plus_2]	France	39
GSE16446[59]	GPL570 [HG-U133_Plus_2]	Europe	107
GSE5327[60]	GPL96 [HG-U133A]	USA	58
GSE12093[61]	GPL96 [HG-U133A]	Europe	136
E-MTAB-748[62]	GPL570 [HG-U133_Plus_2]	France	46
GSE2603[63]	GPL96 [HG-U133A]	USA	82
E-TABM-158[64]	GPL4685 [U133AAofAv2]	USA	128
Total			2735

Table S2. Breast Cancer Microarray Datasets - IPP Score Statistics

Microarray	Median	Mean	Skewness	Kurtosis
GSE45255	-1.128	-1.098	0.2773	0.4199
GSE11121	0.9875	0.8371	-0.6926	0.4871
GSE2034	-0.6178	-0.6180	0.01741	0.9525
GSE58644	0.5740	0.5084	-0.3272	0.09223
GSE58812	0.4106	0.3630	-0.2452	-0.06135
GSE6532_KI	0.4617	0.4094	-0.3078	-0.1101
GSE6532_OXF (GPL570)	0.2502	0.2129	-0.2200	0.1978
GSE6532_OXF (GPL96)	-0.01222	-0.08742	-0.3427	0.4334
E-MTAB-365	-0.2760	-0.2621	-0.01658	0.4929
GSE7390	-0.5712	-0.4871	0.4165	0.2521
GSE9195	-0.3690	-0.3192	0.1888	0.001652
GSE17705	-0.05728	-0.1021	-0.02364	0.6851
GSE20685	-0.2469	-0.2282	0.1390	0.1668
GSE19615	-0.5207	-0.4584	0.3838	0.1504
GSE17907	0.2294	0.2106	-0.2175	0.1232
GSE16446	-0.3991	-0.3368	0.3376	-0.07276
GSE5327	0.1703	0.1135	-0.3026	0.3123
GSE12093	0.2688	0.2089	-0.4207	0.7944
E-MTAB-748	0.1464	0.1095	-0.2655	0.08468
GSE2603	-0.1670	-0.1671	0.01046	0.8123
E-TABM-158	-0.06757	-0.08366	-0.2134	0.3153

Table S3. Breast Cancer Microarray Datasets - Z-Score Statistics from log-rank test with average threshold

Microarray	Median	Mean	Skewness	Kurtosis
GSE45255	-1.259	-1.275	0.09609	0.5463
GSE11121	0.8950	0.7940	-0.2947	1.753
GSE2034	-0.8853	-0.8798	0.1247	0.1652
GSE58644	0.5946	0.5419	-0.2336	0.1459
GSE58812	0.3913	0.3674	-0.1226	-0.1642
GSE6532_KI	0.3439	0.3483	-0.03289	0.03302
GSE6532_OXF (GPL570)	0.2821	0.2828	-0.06509	0.3026
GSE6532_OXF (GPL96)	0.2799	0.1924	-0.3736	0.1813
E-MTAB-365	-0.1838	-0.1672	-0.006944	1.051
GSE7390	-0.4042	-0.3838	0.08515	-0.2239
GSE9195	-0.4663	-0.4211	0.1724	-0.1177
GSE17705	-0.07373	-0.1210	-0.1212	0.3407
GSE20685	-0.09306	-0.1124	-0.1194	0.1141
GSE19615	-0.6071	-0.5699	0.1692	-0.1925
GSE17907	-0.02517	0.004348	0.08427	0.2710
GSE16446	-0.2754	-0.2401	0.1559	0.1105
GSE5327	0.2346	0.1912	-0.2234	0.07154
GSE12093	-0.03932	-0.02753	-0.05476	0.5038
E-MTAB-748	0.1412	0.1308	-0.06038	0.07531
GSE2603	-0.08007	-0.1055	-0.1706	0.3959
E-TABM-158	-0.05878	-0.06988	-0.06741	0.1014

Table S4. Breast Cancer Microarray Datasets - Z-Score Statistics from log-rank test with median threshold

Microarray	Median	Mean	Skewness	Kurtosis
GSE45255	-1.125	-1.160	0.02472	0.5463
GSE11121	0.9374	0.8131	-0.7340	2.197
GSE2034	-0.9287	-0.8945	0.4399	1.675
GSE58644	0.6641	0.6047	-0.1352	0.4878
GSE58812	0.4333	0.4075	-0.2585	0.8093
GSE6532_KI	0.2960	0.3261	0.04179	0.07810
GSE6532_OXF (GPL570)	0.2276	0.2672	0.03707	0.7938
GSE6532_OXF (GPL96)	0.3979	0.1907	-0.2861	0.2040
E-MTAB-365	-0.1785	-0.1365	0.2474	1.107
GSE7390	-0.5689	-0.5270	0.1953	0.2962
GSE9195	-0.6080	-0.4570	0.2111	-0.2518
GSE17705	-0.05456	-0.09919	-0.1822	1.721
GSE20685	-0.07795	-0.09758	-0.09136	0.1357
GSE19615	-0.5318	-0.5554	0.3052	-0.007533
GSE17907	-0.09116	-0.06633	0.05539	0.1250
GSE16446	-0.2109	-0.1808	0.2169	0.3899
GSE5327	0.2639	0.1929	-0.1474	0.1779
GSE12093	-0.08356	-0.05553	0.02759	0.8676
E-MTAB-748	0.2004	0.1322	-0.2510	1.274
GSE2603	-0.03989	-0.07496	-0.1034	0.4006
E-TABM-158	0.01443	-0.02285	0.05906	0.9130

Table S5. Arbitrary, random-made Patients Sampling Score Distributions - Statistics

Case	Median	Mean	2% Percentile	98% Percentile	Skewness	Kurtosis
N=20	0.01671	0.01390	-1.591	1.605	-0.01136	-0.3076
N=30	-0.006343	-0.002693	-1.599	1.601	0.01818	-0.1224
N=40	-0.0001231	-0.001515	-1.565	1.585	0.02861	-0.1306
N=50	-0.001393	-0.0005191	-1.597	1.606	0.007714	0.1152
N=60	0.003878	0.007337	-1.553	1.571	0.005343	0.01034
N=80	-0.01116	-0.006856	-1.568	1.543	0.03257	0.01555
N=100	-0.01287	-0.01477	-1.567	1.501	-0.02175	-0.02459
N=150	0.0007158	-0.002575	-1.529	1.526	0.005994	-0.08410
N=200	0.0003425	-0.001587	-1.480	1.508	0.01332	-0.05097
N=250	-0.002506	1.921e-005	-1.523	1.505	0.02751	0.09992

Table S6. Luminal (ER-positive/PR-positive) Breast Cancer - IPP Score Statistics

Microarray	Used Patient Samples Number	Median	Mean	Skewness	Kurtosis
GSE45255	64	-0.9497	-0.8879	0.3941	0.4977
E-MTAB-748	34	0.1914	0.1549	-0.2468	0.1925
E-TABM-158	65	0.4336	0.3974	-0.4442	0.4491
GSE19615	63	-0.6595	-0.6031	0.3526	-0.02121
GSE2603	35	0.3078	0.2929	-0.1879	0.5524
GSE6532_OXF (GPL570)	64	0.04814	-0.005946	-0.2046	0.06981
E-MTAB-365	46	-0.07676	-0.1054	-0.1920	0.3581
GSE6532_KI	39	0.4253	0.3788	-0.2967	0.04745
GSE9195	59	-0.3732	-0.3300	0.2491	-0.01816
Total	469				

Table S7. HER2-enriched (ER-negative/PR-negative/HER2-positive) Breast Cancer - IPP Score Statistics

Microarray	Used Patient Samples Number	Median	Mean	Skewness	Kurtosis
GSE17907	20	-0.04645	-0.005516	0.1728	-0.2565
GSE45255	23	-0.09486	-0.1527	-0.5577	0.7396
Total	43				

Table S8. Triple-negative (ER-negative/PR-negative/HER2-negative) Breast Cancer - IPP Score Statistics

Microarray	Used Patient Samples Number	Median	Mean	Skewness	Kurtosis
E-TABM-158	21	-0.6327	-0.6508	-0.1101	0.01136
GSE19615	28	-0.5180	-0.4646	0.3704	-0.02457
GSE2603	25	0.03343	0.02830	-0.08825	0.1924
E-MTAB-365	20	-0.2194	-0.1746	0.2277	-0.06568
GSE58812	107	0.4106	0.3631	-0.2451	-0.06253
Total	201				

References

17 Wang, Y. *et al.* Gene-expression profiles to predict distant metastasis of lymph-node-negative primary breast cancer. *Lancet* **365**, 671-679, doi:10.1016/S0140-6736(05)17947-1 (2005).

47 Nagalla, S. *et al.* Interactions between immunity, proliferation and molecular subtype in breast cancer prognosis. *Genome Biology* **14**, doi:10.1186/gb-2013-14-4-r34 (2013).

48 Schmidt, M. *et al.* The humoral immune system has a key prognostic impact in node-negative breast cancer. *Cancer Research* **68**, 5405-5413, doi:10.1158/0008-5472.Can-07-5206 (2008).

49 Tofigh, A. *et al.* The Prognostic Ease and Difficulty of Invasive Breast Carcinoma. *Cell Reports* **9**, 129-142, doi:10.1016/j.celrep.2014.08.073 (2014).

50 Jezequel, P. *et al.* Gene-expression molecular subtyping of triple-negative breast cancer tumours: importance of immune response. *Breast Cancer Research* **17**, doi:10.1186/s13058-015-0550-y (2015).

51 Loi, S. *et al.* Definition of clinically distinct molecular subtypes in estrogen receptor-positive breast carcinomas through genomic grade. *Journal of Clinical Oncology* **25**, 1239-1246, doi:10.1200/Jco.2006.07.1522 (2007).

52 Guedj, M. *et al.* A refined molecular taxonomy of breast cancer. *Oncogene* **31**, 1196-1206, doi:10.1038/onc.2011.301 (2012).

53 Desmedt, C. *et al.* Strong time dependence of the 76-gene prognostic signature for node-negative breast cancer patients in the TRANSBIG multicenter independent validation series. *Clinical Cancer Research* **13**, 3207-3214, doi:10.1158/1078-0432.CCR-06-2765 (2007).

54 Loi, S. *et al.* Predicting prognosis using molecular profiling in estrogen receptor-positive breast cancer treated with tamoxifen. *Bmc Genomics* **9**, doi:10.1186/1471-2164-9-239 (2008).

55 Symmans, W. F. *et al.* Genomic Index of Sensitivity to Endocrine Therapy for Breast Cancer. *Journal of Clinical Oncology* **28**, 4111-4119, doi:10.1200/Jco.2010.28.4273 (2010).

56 Kao, K. J., Chang, K. M., Hsu, H. C. & Huang, A. T. Correlation of microarray-based breast cancer molecular subtypes and clinical outcomes: implications for treatment optimization. *Bmc Cancer* **11**, doi:10.1186/1471-2407-11-143 (2011).

57 Li, Y. *et al.* Amplification of LAPTM4B and YWHAZ contributes to chemotherapy resistance and recurrence of breast cancer. *Nature Medicine* **16**, 214-U121, doi:10.1038/nm.2090 (2010).

58 Sircoulomb, F. *et al.* Genome profiling of ERBB2-amplified breast cancers. *Bmc Cancer* **10**, doi:10.1186/1471-2407-10-539 (2010).

59 Desmedt, C. *et al.* Multifactorial Approach to Predicting Resistance to Anthracyclines. *Journal of Clinical Oncology* **29**, 1578-1586, doi:10.1200/Jco.2010.31.2231 (2011).

60 Minn, A. J. *et al.* Lung metastasis genes couple breast tumor size and metastatic spread. *Proc Natl Acad Sci U S A* **104**, 6740-6745, doi:10.1073/pnas.0701138104 (2007).

61 Zhang, Y. *et al.* The 76-gene signature defines high-risk patients that benefit from adjuvant tamoxifen therapy. *Breast Cancer Res Tr* **116**, 303-309, doi:10.1007/s10549-008-0183-2 (2009).

62 Bonnet, F. *et al.* An array CGH based genomic instability index (G2I) is predictive of clinical outcome in breast cancer and reveals a subset of tumors without lymph node involvement but with poor prognosis. *Bmc Medical Genomics* **5**, doi:10.1186/1755-8794-5-54 (2012).

63 Minn, A. J. *et al.* Genes that mediate breast cancer metastasis to lung. *Nature* **436**, 518-524, doi:10.1038/nature03799 (2005).

64 Chin, K. *et al.* Genomic and transcriptional aberrations linked to breast cancer pathophysiologies. *Cancer Cell* **10**, 529-541, doi:10.1016/j.ccr.2006.10.009 (2006).
